# Polyphenolic Compounds of *Crataegus* Berry, Leaf, and Flower Extracts Affect Viability and Invasive Potential of Human Glioblastoma Cells

**DOI:** 10.3390/molecules26092656

**Published:** 2021-05-01

**Authors:** Natalia Żurek, Olena Karatsai, Maria Jolanta Rędowicz, Ireneusz Tomasz Kapusta

**Affiliations:** 1 Institute of Food Technology and Nutrition, University of Rzeszow, 4 Zelwerowicza St., 35-601 Rzeszow, Poland; nzurek@ur.edu.pl (N.Ż.); j.redowicz@nencki.edu.pl (M.J.R.); 2 Nencki Institute of Experimental Biology, Polish Academy of Sciences, 3 Pasteur St., 02-093 Warsaw, Poland; o.karatsai@nencki.edu.pl

**Keywords:** cell viability, apoptosis, cancer, *Crataegus*, flavonoids, glioblastoma, phenolic acid, morphological parts

## Abstract

*Crataegus* contains numerous health-promoting compounds that are also proposed to have anti-cancer properties. Herein, we aimed at a contemporaneous evaluation of the effects of polyphenol-rich extracts of berries, leaves, and flowers of six *Crataegus* species on the viability and invasive potential on the highly aggressive human glioblastoma U87MG cell line. The treatment with the extracts evoked cytotoxic effects, with the strongest in the berry extracts. All extracts not only promoted the apoptosis-related cleavage of poly (ADP-ribose) polymerase 1 (PARP1) but also substantially inhibited the activity of pro-survival kinases, focal adhesion kinase (FAK), and protein kinase B (PKB; also known as Akt), thus indicating the suppression of proliferative and invasive potentials of the examined glioblastoma cells. The qualitative and quantitative characterization of the extracts’ content was also performed and revealed that amongst 37 polyphenolic compounds identified in the examined *Crataegus* extracts, the majority (29) was detected in berries; the leaf and flower extracts, exerting milder cytotoxic effects, contained only 14 and 13 compounds, respectively. The highest polyphenol content was found in the berries of *C. laevigata x rhipidophylla x monogyna*, in which flavan-3-ols and phenolic acids predominated. Our results demonstrated that a high content of polyphenolic compounds correlated with the extract cytotoxicity, and especially berries were a valuable source of compounds with anti-cancer potential. This might be a promising option for the development of an effective therapeutic strategy against highly malignant glioblastomas in the future.

## 1. Introduction

In recent years, the massive interest in polyphenolic compounds as substances with potential beneficial health properties resulted in an increase in the number of studies aimed at their identification and quantification, especially in the plants that were used for centuries in traditional medicine [1,2,3,4,5,6]. Among them is hawthorn, *Crataegus* L., of the *Rosaceae* family comprising of about 200 species of deciduous shrubs and trees growing wild in the forests of Europe, North Africa, West Asia, and North America. Native Americans used *Crataegus* berries, flowers, and bark as diuretics for kidney and bladder diseases, to treat stomach pains, and to stimulate appetite, while in Europe, it was used to treat asthma, pharyngitis, and insomnia [6,7]. Today, *Crataegus* berries and flowers are officially recognized as herbal raw materials and have been included in European, American, and Canadian pharmacopeias. Recent research confirmed and extended the possibilities of the traditional use of this plant, among others, to anti-inflammatory [8,9], anti-oxidant [10,11], anti-aging [12], anti-depressant [13], anti-viral, anti-fungal, anti-bacterial [14], hypoglycemic, hepatoprotective [15], gastroprotective [16], immunostimulatory, hypolipidemic, hypocholesterolemic, and anti-atherosclerotic effects [17]. Furthermore, *Crataegus* extracts are considered as putative therapeutic agents for the treatment of hepatocellular carcinoma, breast cancer, melanoma, and colorectal cancer [18,19,20]. Thus, *Crataegus* has a high pro-health potential, which could be used in the prevention and/or the treatment of many diseases, including cancer. Health-promoting hawthorn properties are mainly attributed to the high content of biologically active ingredients, of which the polyphenolic compounds are the most important ones. The most essential polyphenolic constituents so far identified in *Crataegus* are phenolic acids (e.g., chlorogenic, caffeic, ferulic, *p*-coumaric, gallic, syringic acids), flavonoids, and flavonoids glycosides (rutin, quercetin 3-*O*-glucoside, luteolin 7-*O*-glucoside, naringenin, C-hexosides, vitexin, isovitexin, kaempferol 3-*O*-glucuronide, myricitrin), anthocyanidins, proanthocyanidins, and their derivatives (epicatechin, procyanidin dimers, procyanidin trimer, cyanidin, cinchonains) [10,11,18,19,20].

Numerous studies indicated that polyphenolic compounds of the extracts of *Crataegus* and several other plants could be behind the observed anti-cancer effects [19,20,21,22,23]. In order to address this notion, we performed comparative studies on polyphenol-rich *Crataegus* extracts obtained from berries, leaves, and flowers of its six species and simultaneously assessed their impact on the viability, morphology, and invasive potential on the human glioblastoma U87MG cell line derived from an aggressive primary malignant brain tumor. All the examined extracts were analyzed for content and chromatographic profiles of secondary metabolites in order to correlate the levels of the particular group of compounds with the observed cellular effects. To date, it has been the first such contemporaneous examination of the effects of *Crataegus* extracts on the glioblastoma cells with the use of both cell biology and phytochemistry techniques.

## 2. Results

### 2.1. Crataegus Extracts Inhibit U87MG Human Glioblastoma Cells Viability

In the light of the reports describing potential anti-tumor properties of *Crataegus* extracts, we decided to examine the effects of its six species growing in the Podkarpacie region on glioblastoma cells derived from a highly malignant brain tumor. The extracts obtained from berries, leaves, and flowers (CB1–6, CL1–6, and CF1–6 extracts, respectively) were evaluated.

**Figure 1:**
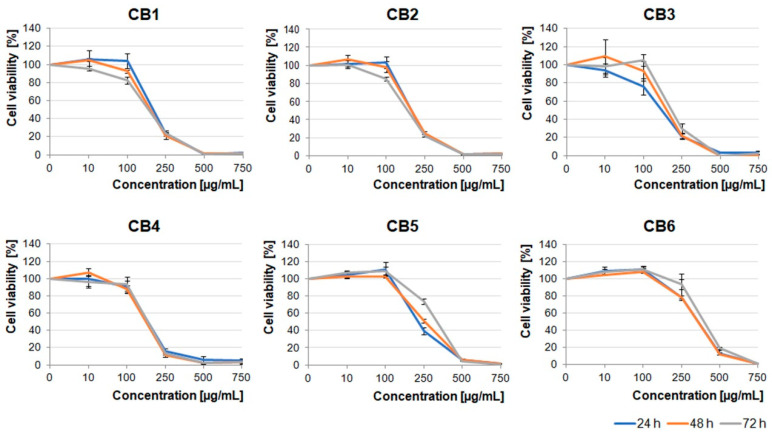
Cytotoxic effect of different concentrations (10–750 µg/mL) of *Crataegus* berry (CB) extracts on U87MG human glioblastoma cells. Cell viability was assayed by the MTS test. Glioblastoma cells were treated up to 72 h with increasing concentrations of the extracts. The number of viable control (non-treated) cells at each time point served as 100%. Graphs represent mean values ± SD from three independent experiments.

**Figure 2:**
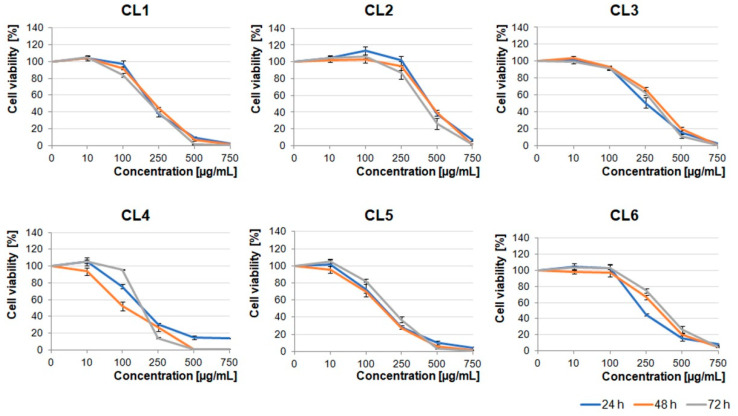
Effect of different concentrations (10–750 µg/mL) of *Crataegus* leaf (CL) extracts on U87MG cell viability. Glioblastoma cells were treated with increasing concentrations of the extracts up to 72 h. The number of viable control (non-treated) cel ls at each time point served as 100%. Graphs represent mean values ± SD from three independent experiments.

**Figure 3:**
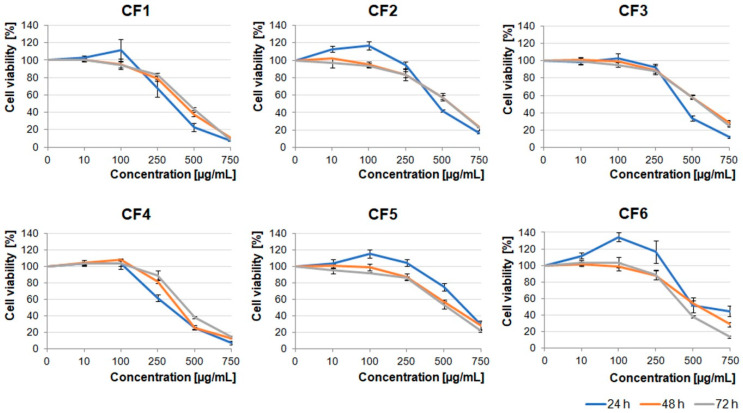
Effect of *Crataegus* flowers (CF) extracts on U87MG cell viability. Glioblastoma cells were treated up to 72 h with increasing concentrations (10–750 µg/mL) of *Crataegus* flower extracts. The number of viable control (non-treated) cells at each time point served as 100%. Graphs represent mean values ± SD from three independent experiments.

First, we analyzed the cytotoxic effect of increasing concentrations (10–750 µg/mL) of the extracts on the U87MG human glioblastoma cell line, which was characterized by a high invasive potential. As shown in Figure 1, Figure 2 and Figure 3, all the examined types of extracts inhibited glioblastoma cell viability, mainly in the concentration-dependent manner. Moreover, berry extracts showed higher cytotoxic effects on glioblastoma cells than the other extracts (Figure 1). A dramatic decrease of cell viability was observed under 250 µg/mL concentration of CB1–CB5 extracts. For CB6, a similar effect was evoked by a higher concentration of 500 µg/mL. The EC50 values for each extract type are presented in Table S1.

*Crataegus* leaf extracts affected glioblastoma cell viability in a pattern similar to that of the berry extracts (Figure 1 and Figure 2). It should be noted that CL1, CL4, and CL5 were more toxic than other leaf extracts (Figure 2).

Extracts obtained from the flowers were less toxic than the ones obtained from berries and leaves. The U87MG cell viability was significantly decreased under concentrations higher than 500 µg/mL (Figure 3). It should be noted that CF1, CF2, CF5, and CF6 in lower concentrations (≤100 µg/mL) stimulated cell proliferation after 24 h incubation. Cells treated with dimethyl sulfoxide (DMSO), in which these extracts were dissolved (0.02–1.5% dependent on the extract concentration), served as an additional negative control (Figure S1).

### 2.2. Do Crataegus Extracts Promote Apoptosis of U87MG Human Glioblastoma Cells?

**Figure 4:**
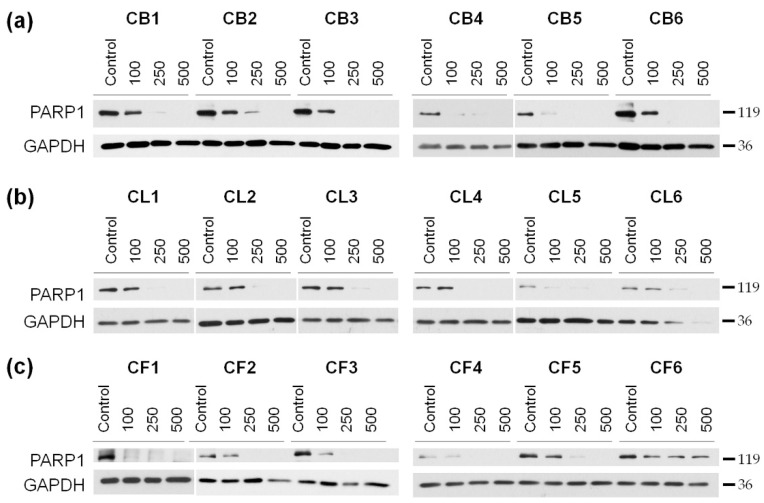
Western blot analysis for the cleavage of poly (ADP-ribose) polymerase 1 (PARP1) in the cell lysates obtained after 48 h of treatment with *Crataegus* berry (**a**), leaf (**b**), and flower (**c**) extracts in 100, 250, and 500 µg/mL concentrations. Non-treated cells served as a control. GAPDH ((glyceraldehyde-3-phosphate dehydrogenase) was used as a protein loading control.

Our next step was to reveal mechanisms of the toxic effects of the extracts on the examined cells. The cells were treated for 48 h with the extract concentrations of 100, 250, and 500 µg/mL, and then the resulted lysates were subjected to the Western blotting to determine the level of poly (ADP-ribose) polymerase 1 (PARP1), which enzymatic cleavage is the hallmark of apoptosis. We showed the concentration-dependent decrease of the full-length PARP1 (Figure 4 and Figure S2) during the treatment with *Crataegus* extracts, thus, indicating that the extracts could promote apoptotic cell death.

### 2.3. Crataegus Extracts Promote Apoptosis-Associated Changes in Morphology of U87MG Human Glioblastoma Cells

**Figure 5:**
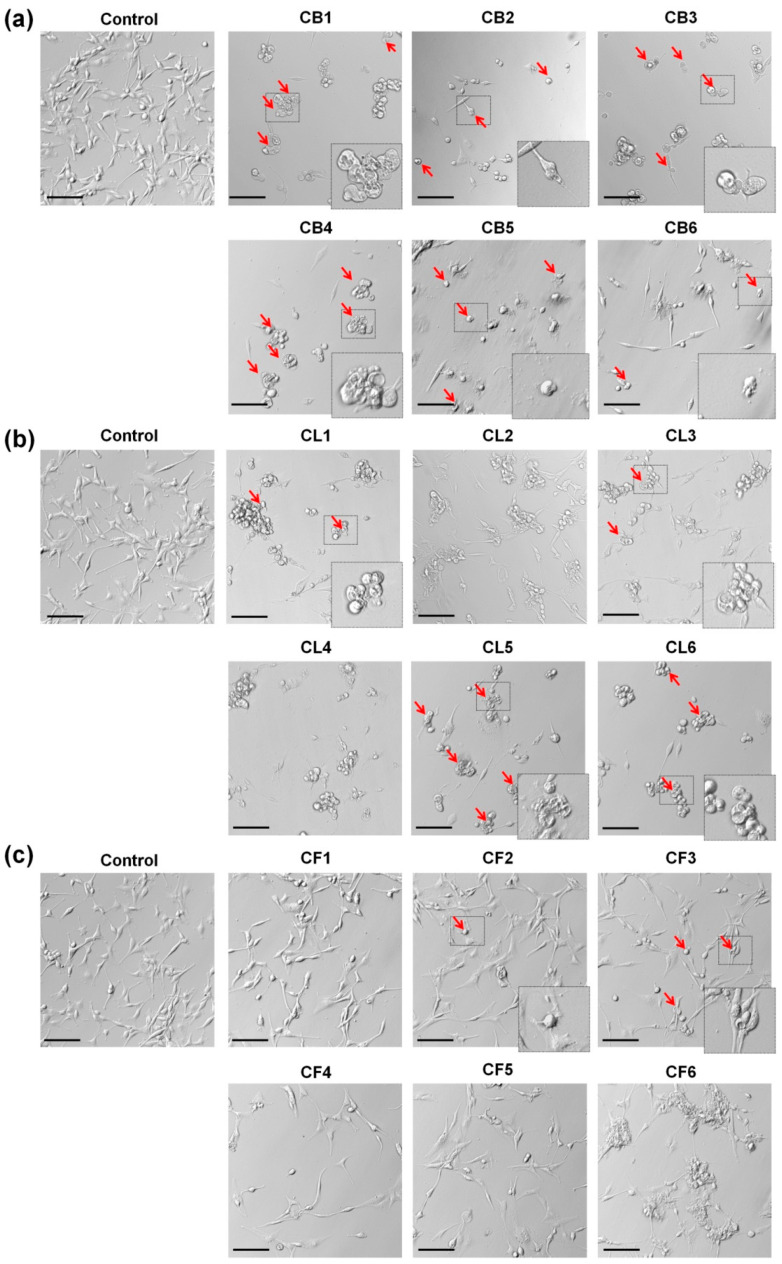
Light microscopy illustrating morphological changes and cell death promotion (red arrows) in U87MG cells after 48 h incubation with the extracts of *Crataegus* berries (**a**), leaves (**b**), and flowers (**c**) at a concentration of 250 µg/mL. Non-treated cells were used as a control for the berry and leaf extracts treatment, and dimethyl sulfoxide (DMSO)-treated cells served as a control for the flower extracts. Bars, 100 μm. Magnification of selected areas, ~2.5×. Arrows point to the cells with apoptotic features.

**Figure 6:**
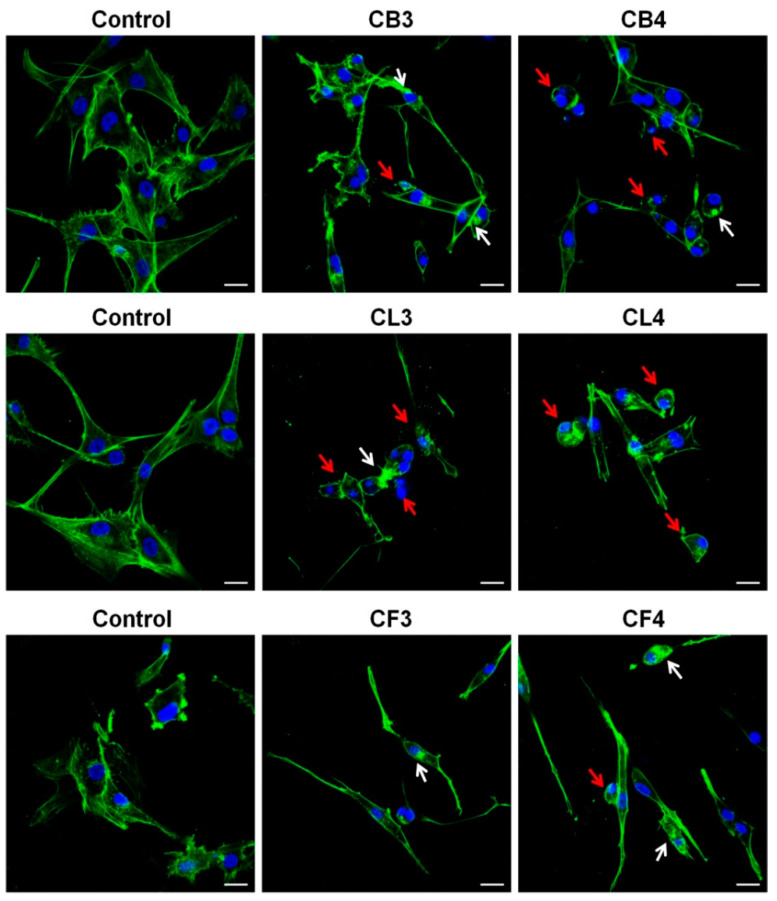
Actin cytoskeleton organization in U87MG cells treated for 48 h with selected *Crataegus* berry (CB3 and CB4), leaf (CL3 and CL4), and flower (CF3 and CF4) extracts at concentrations close to the EC50 values. Non-treated cells served as a control for berry and leaf extracts treatment, and DMSO-treated cells served as a control for flower extracts. Cells were stained with Alexa Fluor 488-conjugated phalloidin (green) and DAPI (blue). Bars, 20 µm. White arrows point to actin aggregates, red arrows point to cells with apoptotic features.

Numerous studies showed that the changes of cell shape, greatly dependent on the cytoskeleton organization, played an important role in cancer cell invasiveness and the progression of cancer metastasis [24,25]. The phenotypic characteristics of glioblastoma cells during a 48 h treatment with *Crataegus* extracted at a concentration of 250 µg/mL (with pronounced effects of most of the examined extracts and close to the EC50 value) were analyzed using light microscopy (Figure 5) and fluorescence staining for the actin cytoskeleton with Alexa Fluor 488-conjugated phalloidin and nuclei with DAPI (Figure 6).

As shown in Figure 5a, all *Crataegus* berry extracts significantly affected the glioblastoma cell morphology. Cells rounded up and did not form lamellipodia, the actin-rich frontal protrusions characteristic of migrating cells. Moreover, the increased number of the cells, with the features of apoptotic cell death such as cell shrinkage and formation of apoptotic bodies (blebs), was observed after the treatment (Figure 5a, red arrows) [26]. Similar effects were observed in the cells treated with CL1, CL5, and CL6 leaf extracts (Figure 5b). In turn, flower extracts had much less pronounced effects on the cell morphology (Figure 5c). Only CF2 and CF3 extracts evoked more evident changes (see Figure 5c, red arrows).

Our next step was a detailed analysis of the impact of *Crataegus* extracts on the actin cytoskeleton and morphology of the examined U87MG cells. For that, we chose the extracts of two *Crataegus* species, *C. x subsphaericea* (CB3, CL3, and CF3) and *C. laevigata x rhipidophylla x monogyna* (CB4, CL4, and CF4), for which we observed the most pronounced cytotoxic effects (Figure 6). For a more precise comparison of *Crataegus* extracts effects, we chose the concentrations close to their EC50 values (see Table S1).

Treatment with CB3 and CB4 berry extracts evoked the loss of cell polarity and a significant decrease of lamellipodia formation in glioblastoma cells that could cause the disturbance in cell motility (Figure 6). Additionally, we observed the formation of actin aggregates, abnormally shaped cell nuclei, and cell rounding, thus, further indicating the progression of apoptotic cell death (Figure 6, see arrows). 

Also, after 48 h incubation with CL3 and CL4 leaf extracts, cells rounded up, and the presence of blebs and actin aggregates was observed (Figure 6, see arrows).

In turn, treatment with CF3 or CF4 flower extracts caused somewhat different changes in glioblastoma cell morphology (Figure 6). Cells became spindle-like, elongated, without a clearly pronounced leading edge. It was noteworthy that an increased level of actin stress fibers in the treated cells was observed. Cells with apoptotic features (shrunk, rounded up, and blebbed) were also present, further confirming the observation that the examined extracts induced apoptosis (Figure 5 and Figure 6, red arrows). 

Thus, treatment with *Crataegus* extracts caused pronounced morphological changes in glioblastoma cells that could affect the invasiveness and viability of the examined cancer cells.

### 2.4. Crataegus Extracts Significantly Decrease the Activity of the Prosurvival FAK and Akt Kinases

Cell adhesion and migration are crucial for cell survival and are essential for cancer cell proliferation and invasiveness [27]. A plethora of proteins are involved in cell attachment to the extracellular matrix (ECM), a three-dimensional network of extracellular macromolecules. Focal adhesion kinase (FAK) is a master regulator of adhesive structure formation and, therefore, is crucial for cell adhesion and motility. It has also been shown that its activity is important for the promotion of cancer cell invasiveness, associated with malignant overgrowth [27,28]. These features make FAK a potential target in the development of anti-cancer treatment.

**Figure 7:**
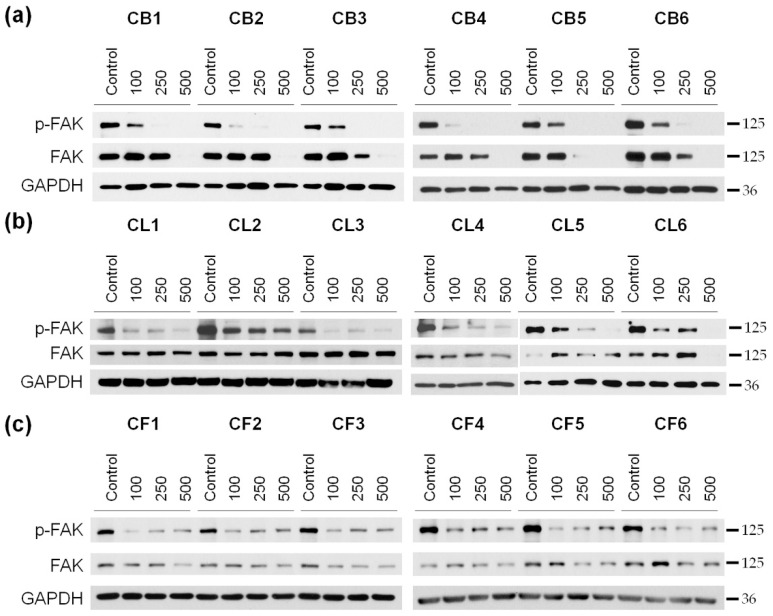
Western blot analysis of the level of focal adhesion kinase (FAK) and its phosphorylated (active) form (p-FAK) in U87MG human glioblastoma cells treated for 48 h with *Crataegus* berry (**a),** leaf (**b**), and flower (**c**) extracts at concentrations of 100, 250, and 500 µg/mL. Non-treated cells served as a control. GAPDH was used as a protein loading control.

We analyzed the level of FAK kinase and its active phosphorylated form (p-FAK) in glioblastoma cells after 48 h of incubation with *Crataegus* extracts (Figure 7 and Figure S3). Since the cytotoxic effect of the extracts was concentration-dependent, three different concentrations (100, 250, and 500 µg/mL) of the extracts were used in the analysis.

We observed that the 48 h treatment with *Crataegus* berry extracts profoundly inhibited the activity of FAK in U87MG cells in a concentration-dependent manner (Figure 7a and Figure 3a). Moreover, incubation of the cells with berry extracts at the concentration of 500 µg/mL completely blocked FAK expression. A similar effect was observed under the treatment with CL5 and CL6 leaf extracts (Figure 7b and Figure S3b). The effect of CL1, CL3, and CL4 extracts was very profound already at a concentration of 100 µg/mL and was not further affected by the increased concentration. An analysis of the effect of all six flower extracts also revealed a decrease in the activation level of FAK that was observed as early as at a concentration of 100 µg/mL. It did not seem to be concentration-dependent as similar levels of p-FAK were observed at higher extract concentrations (Figure 7c and Figure S3c).

**Figure 8:**
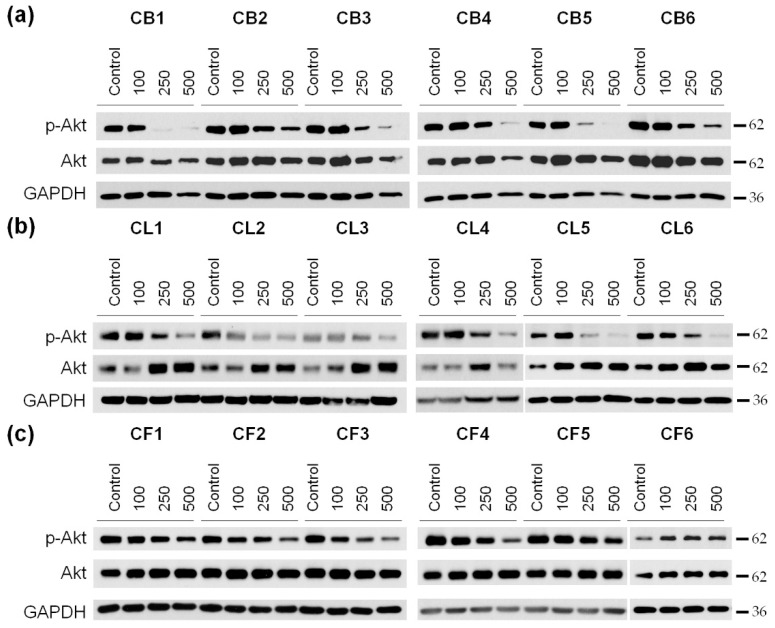
Western blotting of protein kinase B (PKB, also known as Akt) and its phosphorylated (active) form (p-Akt) in U87MG cells treated for 48 h with different concentrations (100, 250, and 500 µg/mL) of *Crataegus* berry (**a**), leaf (**b**), and flower (**c**) extracts. Non-treated cells served as a control. GAPDH was used as a protein loading control.

It is known that cell survival, motility, and cell cycle progression are also highly dependent on the activity of protein kinase B (PKB), also known as Akt [29]. This is why our next step was to examine the phosphorylation (activation) level of Akt in U87MG cells after the incubation with *Crataegus* extracts (Figure 8 and Figure S4). 

A significant decrease in Akt phosphorylation in glioblastoma cells was observed under the cell incubation with nearly all the extracts with the most profound effect of berry extracts, in particular of CB1 (Figure 8a–c and Figure S4a–c). In turn, the 48 h treatment with CF1–CF5 *Crataegus* flower extracts just slightly reduced the level of p-Akt (Figure 8c and Figure S4c). Interestingly, we did not observe significant changes in the p-Akt level during the treatment with CF6, regardless of the increase in its concentration. 

FAK inhibition, together with a decrease in Akt activity upon treatment with the examined extracts, suggested that the extracted compound could profoundly reduce cell growth and migration, and thus, the invasiveness of U87MG glioblastoma cells. 

A better understanding of the mechanisms of the observed effects of *Crataegus* extracts and their individual components should be beneficial for the development of this plant extract-based potential treatment of highly invasive human glioblastomas. Therefore, we aimed at qualitative and quantitative analyses of the content of the examined *Crataegus* extracts.

### 2.5. Identification of Polyphenolic Compounds

It was shown that the health-promoting properties of numerous plant materials, including anti-oxidant, anti-inflammatory, and anti-cancer effects, depended mainly on the content of polyphenols [30]. Therefore, our next step was the analysis for the content of these compounds in the extracts obtained from berries, leaves, and flowers of the examined six species of *Crataegus*. The identification of polyphenolic compounds was performed in the UPLC-PDA-MS/MS system in positive and negative ion mode. The results are summarized in Table 1. The UPLC chromatograms of berries, leaves, and flowers first *Crataegus* species (C1) are presented in Figures S5–S7. A total number of 37 compounds was detected in morphological parts of the examined *Crataegus* species. We found 29 compounds in berries, 14 in leaves, and 13 compounds in flowers. Among all the compounds, four belonged to the anthocyanin category, six to flavan-3-ols, five to hydrolyzable tannins, nine to phenolic acids, and 13 to flavonols. 

Anthocyanins are one of the most important flavonoid pigments found in many plant organs [31]. The anthocyanin profile in *Crataegus* was obtained by detection with the typical absorption maximum for anthocyanins of 520 nm. In total, four compounds were assigned, but only in berries (see Table 1). Their identification was based on fragmentation patterns. Peaks 1 and 3 showed the fragment ion at 287 *m/z* that corresponded to cyanidin. These compounds were identified as cyanidin 3-*O*-glucoside (*m/z* 449) and cyanidin 3-*O*-arabinoside (*m/z* 419), respectively. Peak 2 was assigned as pelargonidin 3-*O*-rutinoside based on the mass spectrum detected at 579 *m/z* and the characteristic fragment ions with *m/z* 271 and 433 (loss of rhamnose and rutinose). The last anthocyanin (peak no. 4) with the molecular ion *m/z* 463 and the fragment ion 301 *m/z* was identified as peonidin 3-*O*-glucoside. 

Another group of polyphenolic compounds id flavan-3-ols, a structurally complex subclass of flavonoids, showing a high pro-health potential [32,33]. Six flavan-3-ols were detected in berries, three in leaves, and two in flowers of *Crataegus*. Compounds 7 and 8 were identified as (+)-catechin and (−)-epicatechin (*m/z* 289), based on commercially available compound standards. Peaks 5, 6, and 9 showed an MS/MS fragment ion at *m/z* 289 (epicatechin derived from a cleavage of the link between procyanidin monomers) [34]. These compounds were assigned as B-type procyanidin trimer (*m/z* 865), dimer (*m/z* 577), and tetramer (*m/z* 1442), respectively. In turn, compound no. 10, representing the parent ion at *m/z* 451 belonging to flavalignan isomers groups, was identified as a cinchonain [35]. 

Hydrolyzable tannins form another group that we identified in *Crataegus* extracts, including five compounds in the berries and one in leaves. Tannins possess well-characterized but opposite activities, ranging from favorable anti-oxidants to harmful pro-oxidants [36]. Peak no. 11 was assigned to the group of the glycosylated form of ellagic acid [37]. Thus, this compound at *m/z* 433 and fragment of *m/z* 301 (loss of a hexose) was identified as ellagic acid pentoside. Compounds 12 and 13 possessed the same molecular ion *m/z* 781 and fragment ion *m/z* 299, that corresponded to the punicalin isomer (α/*β*-isomer) [38]. Since the *β*-isomer eluted before the α-isomer, peak 12 was identified as *β*-isomer and peak 13 as α-isomer (isopunicalin). The last two hydrolyzable tannins (peak no. 14 and 15) with *m/z* 721, 739, and *m/z* 301 ion fragments assigned to bis-HHDP-glucose, belonging to galloyl substituents, were detected as 2-*O*-galloylpunicalin (*m/z* 933) and eucalbanin A (*m/z* 1085), respectively [39].

Phenolic acids belong to the next main group of polyphenolic compounds detected in all food products and present both in free and conjugated forms. Among the analyzed parts of *Crataegus*, six phenolic acids were identified in berries, five in leaves, and five in flowers. Compound 16 detected at *m/z* 191 was identified as quinic acid according to the standards. Two peaks, no. 17 and 21, had fragment ions *m/z* 163 typical for *p*-coumaric and *m/z* 119, corresponding to decarboxylated form coumaric acid. Therefore, these two peaks were tentatively annotated as coumaroylquinic acid and 3-*O*-*p*-coumaroylquinic acid, respectively. Peaks no. 19 and 22 represented acylated quinic acid derivatives, including chlorogenic acids, with the ion at *m/z* 353 and a fragment *m/z* 191 (quinic acid) typical of caffeoylquinic acid were identified as 4-*O*-caffeoylquinic acid and 3-*O*-caffeoylquinic acid. Compound no. 24 identified as 3,4-*O*-dicaffeoylquinic acid (515 *m/z*), with the fragmentation ion at *m/z* 353 also belonging to this category. Compound 18 with *m/z* of 315 and the produced ion at *m/z* 153, corresponding to protocatechuic acid after the neutral loss of the hexoside group, was identified as protocatechuic acid glucoside. The other two phenolic acids compounds 20 (*m/z* 297) and 23 (*m/z* 281), were assigned to unidentified caffeic and cumaric derivatives, respectively.

Flavonols are a large class of flavonoids, widely distributed within the plant kingdom and present in high concentrations in the epidermis of leaves and fruit skin. Eight compounds of this category were detected in berries, four in leaves, and six in flowers of *Crataegus*. Among flavonols, three compounds (no. 25, 26, 27) with the same molecular weight occurred in berries. These compounds were tentatively identified based on the primary ion at *m/z* 787 and MS/MS fragmentation, giving the ion at *m/z* 421 through the loss of galloyl moiety and gallic acid, respectively; thus, were identified as tetra-*O*-galloyl-glucosides. Peak no. 29 (*m/z* 609) produced a fragmentation ion at *m/z* 285, belonging to luteolin derivatives, was identified as luteolin 6,8-C-diglucoside. Analyzed compounds 28, 36, and 37 with ions *m/z* of 431, 563, and 619, characteristic for apigenin derivatives, were tentatively identified as apigenin 8-C-glucoside (vitexin), apigenin 6-C-glucoside-8-C-arabinoside, and cratenacin, respectively. Based on the standards, four quercetin derivatives (fragment ion *m/z* 301) were detected such as: quercetin, then 3-*O*-rutinoside (no. 30, *m/z* 609), quercetin 3-*O*-glucoside (no. 32, *m/z* 463), quercetin 3-*O*-galactoside (no. 33, *m/z* 463), and quercetin 3-*O*-acetyl hexoside (no. 34, *m/z* 505). The next two peaks, 31 (*m/z* 433) and 35 (*m/z* 463), were assigned as naringenin 7-*O*-glucoside and myricetin 3-*O*-rhamnoside, respectively. The assignment was based on the presence of the characteristic fragment ions at *m/z* 271 for naringenin and *m/z* 317 for myricetin [40].

### 2.6. Quantification of Polyphenolic Compounds

**Figure 9:**
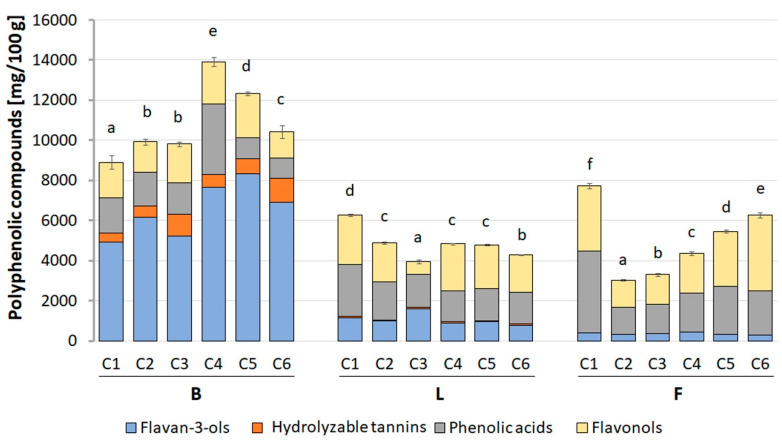
The content of polyphenolic compounds in extracts of berries, leaves, and flowers of six examined species of *Crataegus*. The graph represents the mean values ± SD from three independent experiments. The statistical significance was analyzed using Duncan’s test. Values marked with different letters (between the species) within berries, leaves, or flowers indicate statistically significant differences (*p* < 0.05; for further details, see Materials and Methods section). Abbreviations: B, berries; L, leaves; F, flowers; C1–C6, examined *Crataegus* species.

The quantification of polyphenolic compounds was performed with the use of the UPLC-PDA-MS/MS method and quantitative data calculated from the calibration curves. The profile and content of polyphenols differing in the individual morphological parts of the *Crataegus* species are presented in Figure 9 and Table S2. The highest content of polyphenolic compounds was determined in the berries, where the estimated value ranged from 8980.77 (in CB1) to 14,038.21 (in CB4) mg/100 g dry basis (d.b.). In turn, the total content of polyphenolic compounds in flowers and leaves was about twice lower and ranged from 3028.82 to 7718.00 mg/100 g d.b., and 3963.15 to 6263.56 mg/100 g d.b. for CF2 and CF1, and CL3, and CL1, respectively.

Among the examined morphological parts of the six species of *Crataegus*, CB4 berry extracts were characterized by the highest content of polyphenolic compounds, in which approximately 55.1% of all phenols were flavan-3-ols. Their quantity was 7671.94 mg/100 g d.b. and was about 14 and 9 times higher, respectively, with respect to the flowers and leaves of the same *Crataegus* species. The dominant compounds were procyanidin dimer (2578.00 mg/100 g d.b.), procyanidin trimer (2000.45 mg/100 g d.b.), and (−)-epicatechin (1800.44 mg/100 g d.b.). The other categories of polyphenolic compounds were present in CB4 berries in the following order: phenolic acids (25.1%) > flavonols (15.2%) > hydrolyzable tannins (4.5%) > anthocyanins (0.1%).

As indicated above, the flower and leaf extracts with the highest total content of polyphenolic compounds were derived from the first species of *Crataegus* (C1). In these morphological parts, the most abundant compounds were phenolic acids, which constituted 52.5% and 41.4% of all CF1 flower and CL1 leaf phenols, respectively. Their amount was 4052.36 and 2591.63 mg/100 g d.b. and was about 4 and 2 times higher with respect to the CB1 berries. The most dominant compounds in CF1 flower extract were 3-*O*-caffeoylquinic acid (2588.40 mg/100 g d.b.), 3-*O*-*p*-coumaroylquinic acid (551.02 mg/100 g d.b.), and 3,4-*O*-di-caffeoyl-quinic acid (458.56 mg/100 g d.b.). In the CL1 leaf extract, the content of particular compounds was: 3-*O*-caffeoylquinic acid (1463.94 mg/100 g d.b.), unidentified caffeic derivative (441.64 mg/100 g d.b.), and 3-*O*-*p*-coumaroylquinic acid (357.04 mg/100 g d.b.). The quantitative profile of the other categories of polyphenolic compounds present in CF1 flowers and CL1 leaves were as follows: flavonols (42.1% and 39.1%, respectively) > flavan-3-ols (5.4% and 18.4%) > hydrolyzable tannins (0% and 1.2%).

## 3. Discussion

Despite the fact that in recent years progress in the treatment of glioblastomas has been made, these malignant brain tumors are still one of the most deadly and most difficult to treat in all oncology [41]. These most common primary brain tumors account for nearly 70% of oligodendroglial, and astrocytic tumors are characterized by a rapid clinical course, poor response rates, high invasiveness, and short survival time, on average from 12 to 15 months since diagnosis [42]. Thus there is the need to search for new, alternative, and/or complementary therapies that could improve the prevention and/or treatment of glioblastomas. 

*Crataegus* is one of the best known medicinal plants with a high content of biologically active ingredients, the most important of which are polyphenols [30]. These compounds have gained importance in recent years due to their high cytotoxic potential against several cancer cells. It was shown that polyphenol-rich extracts from various anatomical parts of *Crataegus* promoted apoptosis of breast adenocarcinoma (MCF-7) [43], cervical cancer (HeLa), liver cancer (HepG2), neuroblastoma (IMR-32), colorectal adenocarcinoma (SW480, Coco-2) [44,45], and melanoma (B16F10) [21]. At the same time, these extracts showed no cytotoxic effects on healthy colon epithelial cells [45] and normal human peripheral mononuclear cells [46]. Additionally, the anti-oxidant activity of the phenolic composition and individual compounds from *Crataegus pinnatifida* berries [47], *Crataegus monogyna* leaves [48], and *Crataegus oxyacantha* flowers and berries [49] were described. The recent reports showed mild cytotoxic effects of phenolic acids from *Petroselinum crispum* L. on human U87MG cells [22], flavonoids from *Lippa graveolens* on human U251MG cells [23], and anthocyanins from *Rubus liebmannii* and *Rubus palmeri* on C6 and RG2 rat and murine, respectively, glioma cell lines [50]. However, there were no studies performed at the cellular level, and all the morphological parts of *Crataegus* were tested in the same set of experiments.

In this work, we performed, for the first time, a thorough contemporaneous screening of the anti-cancer potential of six *Crataegus* species, i.e., we examined whether polyphenolic extracts from their berries, leaves, and flowers could affect the viability, morphology, and invasive potential of the well-characterized human glioblastoma U87MG cell line. Moreover, we determined both qualitatively and quantitatively the content of these extracts to identify the species and/or plant organs with the highest anti-cancer potential. It was noteworthy that our polyphenolic extracts were processed with the use of the adsorbing resin (RP-18) that enabled obtaining polyphenol-rich preparations devoid of sugars, proteins, and minerals.

Our results showed that all the examined polyphenolic *Crataegus* extracts inhibited the viability of the examined cells in a dose-dependent manner. However, our data indicated that the level of the observed inhibition did not depend on the incubation time, as prolonged incubation (up to 72 h) had little or any effect on the treated cells, indicating that the extract compounds could target the vital processes relatively fast. We also demonstrated that the berry extracts evoked the strongest cytotoxic activity in comparison with the leaf and flower extracts.

This differential effect of the extracts on U87MG cells was most likely related to the distinctive classes of polyphenolic components found in anatomical parts of the examined *Crataegus* species. Phytochemical analysis revealed that, among the identified and quantified polyphenolic compounds, the highest number (29 out of 37 in total) was found in berries, mainly in the CB4 sample, which also showed the strongest inhibitory effect on U87MG cell viability. Noticeably, the berries were the only organs containing anthocyanins, though in relatively small amounts (<0.1%). The most abundant group within the berry extracts was flavan-3-ols, including flavonoid oligomers such as procyanidin dimer and procyanidin trimer. Reports published so far by other groups showed that purified procyanidins inhibited cell viability and/or induced cell apoptosis of breast cancer cells (MCF-7 and MDA-MB-468 lines), lung cancer (A427 lines), prostate cancer (DU145 line), colorectal cancer (HCT-8, HT29, and Caco-2 lines) and bladder cancer (BIU87 lines) without a cytotoxic effect on untransformed counterparts [51]. It was also shown that the chemical structure of polyphenols was a decisive factor in their anti-cancer effectiveness, and, in the case of oligomeric flavan-3-ols, their degree of polymerization positively correlated with the reduced viability of neoplastic cells [52]. 

Our UPLC analysis also showed that the CB4 sample, derived from berries of *C. laevigata x rhipidophylla x monogyna*, compared to the berry extracts from other species, contained approximately 3 times more phenolic acids, in particular of 4-*O*-caffeoylquinic acid and 3-*O*-caffeoylquinic acid. This group of compounds was shown to have cytotoxic effects on breast adenocarcinoma cells (MCF-7) [43], melanoma cells (HCT15), colon adenocarcinoma (HT29) [53], and lung carcinoma (NCI-H23) [52]. Presumably, the relationship between the structure and anti-tumor activity was also applied to these compounds. It was also proposed that esterification of the carboxyl group of caffeic acid with quinic acid could affect the biological effectiveness of these derivatives [54].

Therefore, the high content of phenolic acids and flavan-3-ols in the examined *Crataegus* berries could explain their strongest effect on U87MG cells. However, it cannot be ruled out that the high biological activity of the *Crataegus* berry extracts could also result from the synergistic effect of polyphenols present in this plant organ. This notion seemed to be confirmed by the studies demonstrating that the effect of the combination of two polyphenolic acids on the anti-proliferative activity of melanoma cells was more pronounced than the mono treatment [55]. It is noteworthy that combination therapy is known to be more effective in treating cancers as it reduces the chances of developing drug resistance. It was shown, among others, that polyphenolic compounds apart from the mentioned mutual combinations could also be combined with other natural compounds such as sugar derivatives, glycosides, peptides, and amino acids [51]. These studies seemed to be highly relevant in the context of our recent report showing that plant-derived amino acid canavanine (an arginine analog) had a strong cytotoxic effect on two human glioblastoma cell lines, U87MG and U251MG [56].

To better understand the mechanisms of the observed cytotoxicity triggered by the extracts, we examined the level of the apoptosis-related PARP1 protein. The cleavage of PARP1, mainly by the executive caspases, led to irreversible DNA damage and played an important role in the regulation of cell death as well as was considered to be a hallmark of ongoing apoptosis. In our study, we observed that *Crataegus* extracts promoted PARP1 cleavage, thus indicating induction of processes leading to apoptotic cell death. PARP1 cleavage was accompanied by morphological aberrations and changes in the cytoskeleton organization characteristic for apoptosis. In particular, treatment of the cells with *Crataegus* berry extracts led to cell shrinkage, blebbing, and changes in the nuclei shape. The treatment also affected the FAK and Akt signaling pathways that were known to control cell proliferation, invasiveness, and cell cycle progression. The observed concentration-dependent decrease in the activity of FAK and Akt kinases upon the treatment were most pronounced for berry extracts, further confirming their potential of being used in the development of a potential anti-glioblastoma treatment.

Summarizing, we compared for first time effects of the extracts of berries, leaves, and flowers of six species of *Crataegus* on human glioblastoma U87MG cells and showed that the berry extracts, in particular, that of *C. laevigata x rhipidophylla x monogyna*, had the strongest cytotoxic effects. Namely, the substantial decrease of cell viability, the induction of the processes leading to apoptotic cell death, and inhibition of the prosurvival signaling pathways that were observed. We suggest that these effects could be associated with the high content of flavan-3-ols and phenolic acids in the berry extracts. The data presented herein could serve in the future as the ground for the development of a potential anti-glioblastoma therapeutical strategy based either on a single compound or the multi-compound combination.

## 4. Materials and Methods

### 4.1. Materials and Reagents

CellTiter 96^®^ AQueous Non-Radioactive Cell Proliferation Assay was purchased from Promega (G5421; Madison, WI, USA). Alexa Fluor 488-conjugated phalloidin was from Invitrogen (A12379; Waltham, MA, USA). Vectashield anti-fade reagent with DAPI was obtained from Vector Laboratories (H-1200; Burlingame, CA, USA). The following antibodies were used: against PARP1 (#9532), FAK (#3285) and p-FAK (#8556), Akt (#9272) and p-Akt (#9271; Cell Signaling Technologies; Danvers, MA, USA), and GAPDH (MAB374; Millipore, St. Louis, MO, USA). Secondary antibodies were from Millipore (USA): HRP-conjugated anti-mouse (AP308P) and anti-rabbit IgG (AP307P). Methanol (64860), acetonitrile (64851), dimethyl sulfoxide (DMSO, D2438), LiChroprep RP-18 (40–63 µm) (1.13900) were purchased from Sigma-Aldrich (Steinheim, Germany). Chlorogenic acid (#4991S), caffeic acid (#6034S), kaempferol 3-*O*-glucoside (#1243S), quercetin 3-*O*-diglucoside (#1347S), quercetin-3-*O*-galactoside (#1027S), quercetin 3-*O*-rutinoside (#1139S), naringenin 7-*O*-glucoside (#1160S), myricetin 3-*O*-rhamnoside (#1029S), pelargonidin 3-*O*-rutinoside (#0943), peonidin 3-*O*-glucoside (#0929S), apigenin 8-*O*-glucoside (#1232S), quinic acid (#0762A), (+)-catechin (#0796S), and (−)-epicatechin (#0977S) were obtained from Extrasynthese (Lyon, France). Cyanidin 3-*O*-glucoside chloride (1151935USP) and punicalin (A + B mixture) (PHL83532) were purchased from Sigma-Aldrich (Steinheim, Germany).

### 4.2. Plant Material

The material derived from six hawthorn species (*Crataegus*), namely C. *monogyna*, *C. rhipidophylla*, *C. x subsphaericea*, *C. laevigata x rhipidophylla x monogyna*, *C. macrocarpa*, and *C. laevigata*, collected by Dr. Mateusz Wolanin from the Department of Botany of the Institute of Biology and Biotechnology, University of Rzeszów, was used in the study. Flower (F) and leaf (L) samples were collected in May, and berries (B) in October at the Błażowa and Piątkowa near Rzeszow (Poland). Characteristics of the selected plants are presented in Table 2. The collected material was lyophilized (ALPHA 1-2 LD plus, Martin Christ Gefriertrocknungsanlagen GmbH, Osterode am Harz, Germany), then ground with a coffee grinder, and stored at −20 °C until preparation of the extracts.

### 4.3. Extracts Preparation

Powdered berries, leaves, and flowers (30 g of each) of six *Crataegus* species were suspended in 300 mL of acetone (50%; *v*/*v*) and left in a shaded place at room temperature (RT) for 24 h. After that, each obtained suspension was centrifuged at 12,000× *g* for 10 min (Centrifuge 5430, Eppendorf, Hamburg, Germany), decanted, and the pellets were re-extracted with acetone (70%; *v*/*v*) using ultrasound (Sonic 10 ultrasonic bath, Polsonic, Warsaw, Poland) at 30 °C for 30 min. After centrifugation (12,000× *g* for 10 min), the supernatants were combined and pre-concentrated using a rotary evaporator at 40 °C (R-215 Rotavapor System, Buchi, Switzerland). The obtained samples were then applied to a column containing adsorber LiChroprep RP-18 (40–63 µm; previously preconditioned with absolute methanol and equilibrated with distilled water). The column was first washed with water to remove polar compounds, and then polyphenols compounds were eluted with methanol (99.8%). The eluates were collected, evaporated, lyophilized, and stored at −20 °C until further analysis. For biological assays, *Crataegus* berries and leaf extracts were dissolved in water, and *Crataegus* flower extracts were dissolved in DMSO and diluted in the cell culture medium prior to the experiments. The yield of the particular samples is presented in Table S3.

### 4.4. Cell Culture

U87MG human glioblastoma cell line was obtained from the Cell Lines Service (Eppelheim, Germany). Cells were cultured at 37 °C in the atmosphere with 5% CO_2_, in Dulbecco’s Modified Eagle Medium-GlutaMAX-1 (DMEM; Gibco 31966021, Gibco, Waltham, MA, USA), supplemented with 10% heat-inactivated fetal bovine serum (FBS; Gibco 10500064, Gibco, Waltham, MA, USA), and antibiotics (100 U/mL penicillin, 100 U/mL streptomycins; Gibco 15140122, Gibco, Waltham, MA, USA). For the passaging, cells were washed by phosphate-buffered saline (PBS) and trypsinized (0.25% trypsin-EDTA; Gibco 25200056, Gibco, Waltham, MA, USA).

### 4.5. MTS Cell Viability Assay

Cells were seeded into 96-well microplates (Greiner, Kremsmünster, Austria) at a density of 8 × 10^3^ cells/well and then incubated at 37 °C. After 18 h incubation, the medium was changed into the experimental conditions. Five concentrations of *Crataegus* extracts were used: 10, 100, 250, 500, and 750 µg/mL. After 24, 48, and 72 h of incubation, the MTS test was performed according to the manufacturer protocol (Promega, Madison, WI, USA). The absorbance was measured at 490 nm using a microplate reader (Sunrise Microplate Reader Remote-Elisa Assays, Tecan Trading AG, Männedorf, Switzerland), and cell viability was estimated as a percentage relative to control (untreated cells, 100%).

### 4.6. Western Blot Analysis of the Cell Lysates

Cells were seeded at a density of 3 × 10^5^ cells per 6-cm culture dish (Sarstedt, Nümbrecht, Germany). After 18 h of culture, cells were treated with *Crataegus* extracts at three concentrations of 100, 250, and 500 µg/mL for 48 h. After incubation, cells were washed twice with ice-cold PBS and lysed in RIPA buffer (50 mM Tris-HCl, pH 7.5, 150 mM NaCl, 0.1% SDS, 0.5% sodium deoxycholate, 1% IGEPAL, 50 mM NaF) supplemented with 2 mM Na_3_VO_4_, 1 mM PMSF, phosphatase, and protease inhibitors (04906837001 and 04693116001, respectively; Roche, Mannheim, Germany) in 4 °C for 20 min. Cell lysates were received after centrifugation at 13,500× *g* at 4 °C for 20 min, and supernatants were collected. The protein concentration was determined according to the Bradford assay (Bio-Rad, Hercules, CA, USA, USA). Next, cell lysates were mixed with the Laemmli buffer (0.25 M Tris-HCl, pH 6.8, 50% glycerol, 5% SDS, 5% *β*-mercaptoethanol, 0.05% bromophenol blue) and incubated for 5 min at 98 °C. Cell proteins were separated in 10 or 12% sodium dodecyl sulfate polyacrylamide gels (SDS-PAGE) and transferred to a nitrocellulose membrane (Amersham 10600002, Freiburg, Germany). Subsequently, membranes were blocked by 5% bovine serum albumin (BSA) solution or 3–5% fat-free milk solution in 0.2% TBS-T (Tris-buffered saline containing 0.2% Triton X-100) for 1 h, and incubated with primary antibodies with PARP (1:750), FAK (1:1000), p-FAK (1:1000), Akt (1:1000), and p-Akt (1:1000) overnight at 4 °C; with GAPDH (1:20,000) for 30 min at the room temperature (RT), and then with secondary antibodies (1:10,000) for 1 h at RT. GAPDH was used as a protein loading control. Protein bands were detected using ECL substrate (Millipore, St. Louis, MO, USA or Termo Fisher Scientific, Waltham, MA, USA). Then, band densitometry was quantified using the Fiji distribution of the ImageJ 1.52a software (National Institutes of Health, Bethesda, MD, USA, and the University of Wisconsin, Madison, WI, USA). The level of GAPDH was used as a protein loading control.

### 4.7. Light Microscopy Analysis 

Cells were seeded at a density of 3 × 10^5^ cells per 6-cm culture dish (Sarstedt, Nümbrecht, Germany). After 18 h, cells were treated with *Crataegus* extracts (250 µg/mL) for 48 h. Non-treated cells were used as a control. Cells incubated with DMSO at concentrations of 0.02–1.5% dependent on the flower extract concentrations (see Figure S1) served as an additional control for the flower extract analysis. Cell morphology was analyzed by light microscopy (Nikon Eclipse Ti-U fluorescent microscope equipped with a 20× objective and DS-Qi2 digital camera, Tokyo, Japan).

### 4.8. Fluorescent Stainings and Confocal Microscopy Analysis 

Cells were seeded in 6-well plates (Sarstedt, Nümbrecht, Germany) with glass coverslips (VWR 631-0153, VWR, Gdańsk, Poland) at a density of 4 × 10^4^ cells/well. After 48 h treatment with *Crataegus* extracts (at concentrations close to the EC50 values), the cells were washed twice with PBS and incubated with 4% paraformaldehyde solution (PFA) at RT for 20 min. Then, the cells were washed with PBS, incubated with a 50 mM NH_4_Cl solution for 30 min, and permeabilized with 0.2% Triton X-100 in PBS for 10 min. Filamentous actin was visualized by staining with Alexa Fluor 488-conjugated phalloidin (1:40 in PBS) for 20 min. Next, the cells were washed with PBS/0.02% Triton X-100 and mounted by Vectashield anti-fade reagent containing DAPI to stain the nuclei. Microphotographs were collected using Zeiss LSM780, Inverted Axio Observer Z.1 with Plan Apochromat 40×/1.4 Oil DIC objective (Carl Zeiss AG, Jena, Germany). Images were prepared using the Zen Blue 2.1 software (Carl Zeiss Microscopy, Jena, Germany).

### 4.9. Determination of Polyphenols Profile

Determination of polyphenolic compounds was carried out using the Ultra Performance Liquid Chromatography (UPLC) Waters ACQUITY system (Waters, Milford, MA, USA). UPLC was equipped with a binary pump manager, column manager, sample manager, photodiode array (PDA) detector, tandem quadrupole mass spectrometer (TQD) with electrospray ionization (ESI) source. Separation of polyphenols was performed using a 1.7 µm, 100 mm × 2.1 mm UPLC BEH RP C18 column (Waters, Milford, MA, USA). For the anthocyanin investigation, the mobile phase consisted of 2% formic acid in water, *v/v* (solvent A), and 2% formic acid, in 40% acetonitrile, *v/v* (solvent B). However, in the case of other polyphenolic compounds, water (solvent A) and 40% acetonitrile, *v/v* (solvent B) were used. The flow rate was kept constant at 0.35 mL/min for a total run time of 8 min. The system was run with the following gradient program: from 0 min 5% B, from 0 to 8 min linear to 100% B, and from 8 to 9.5 min for washing and back to initial conditions. The injection volume of the samples was 5 µL, and the column was supported at 50 °C. The following TQD parameters were used: cone voltage of 30 V, capillary voltage of 3500 V, source and desolvation temperature 120 °C and 350 °C, respectively, and desolvation gas flow rate of 800 L/h. Characterization of the individual polyphenolic compounds was performed on the basis of the retention time, mass-to-charge ratio, fragment ions, and comparison of data obtained with commercial standards and literature findings. Obtained data were processed in Waters MassLynx v.4.1 software (Waters, Milford, MA, USA). Extracts (10 mg of each) of *Crataegus* berries and leaves were dissolved in water, while the flower was extracted in acetonitrile (50%; *v/v*),and then diluted in water in 1:5 ratio. All experiments were done in triplicates, and the results were expressed as mg/100 g of dry basis (d.b.).

### 4.10. Quantification of Polyphenolics and Method Validation

The quantification of polyphenolic compounds was performed by the use of different internal standards (Table S4). Stock solutions of phenolic standards were prepared at 1 mg/mL after dissolving in 50% acetonitrile in water. For quantification of phenolic compounds, the working solutions of mixed analytes at the concentrations of 25, 50, 100, 150, 250 µg/mL were obtained by dilution of the appropriate volume of stock solutions. Concentrations of polyphenolics were calculated by preparing a calibration curve of mass concentration vs. peak area. The slope of the regression line and values of correlation coefficient (R^2^) for each standard curve were obtained using MS Excel 2019 software. Limits of detection (LOD) and limits of quantification (LOQ) were calculated for each sample in triplicates. Calibration curves were obtained consecutively by plotting concentration against the peak area. The mean of the slope (S) and standard deviation of intercept (δ) were calculated from the standard curve of three replicates. LOD and LOQ were calculated with the following Equations:

LOD = 3.3 × (δ/S)(1)

LOQ = 10 × (δ/S)(2)

The intra- and interday variations were determined using relative standard deviation (RSD) values, which were <3.5% for all the analyzed compounds.

### 4.11. Statistics

All experiments were performed at least three times. The results were shown as means ± SD. Statistical significance for polyphenolic compounds was analyzed by one-way analysis of variance (ANOVA) using Duncan’s test. Values marked with different letters indicated statistically significant differences (*p* < 0.05). Values marked with the same letter within the same group did not differ statistically. All calculations were made in Statistica v. 13.3 software (StatSoft, Krakow, Poland). Statistical analyses of the levels of proteins were performed using a one-way ANOVA test in the GraphPad Prism 8.4.3 software (San Diego, CA, USA). Statistical significance was defined as *p* < 0.05.

## Figures and Tables

**Table 1:** Summary of phenolic compounds identified by UPLC-PDA-MS/MS in extracts of berries, leaves, and flowers of the examined *Crataegus* species.

Peak No.	Tentative Assignment	λ_max_ nm	(M − H) *m/z*		*Crataegus*		
			MS	MS/MS	B	L	F
1	Cyanidin 3-*O*-glucoside	278, 514	449^+^	287	+		
2	Pelargonidin 3-*O*-rutinoside	279, 517	579^+^	271, 433	+		
3	Cyanidin 3-*O*-arabinoside	274, 510	419^+^	287	+		
4	Peonidin 3-*O*-glucoside	278, 516	463^+^	301	+		
*Flavan-3-ols*							
5	Procyanidin trimer (B-type)	278	865	289	+	+	
6	Procyanidin dimer (B-type)	279	577	289	+	+	+
7	(+)-Catechin	281	289	-	+	+	+
8	(−)-Epicatechin	274	289	245	+		
9	Procyanidin tetramer (B-type)	278	1442	720[M − 2H]^−2^, 577, 289	+		
10	Cinchonain (A-type)	279, 377	451	341, 176	+		
11	Ellagic acid pentoside	278	433	301	+		
12	Punicalin isomer *β*	279	781	721, 299	+	+	
13	Punicalin isomer α	278	781	299	+		
14	2-*O*-galloylpunicalin	279	933	721, 301	+		
15	Eucalbanin A	279	1085	739, 301	+		
16	Quinic acid	274	191	173	+		
17	Coumaroylquinic acid	309	337	163, 119	+		
18	Protocatechuic acid glucoside	251sh, 288	315	153	+		
19	4-*O*-caffeoylquinic acid	299sh, 324	353	191	+		
20	Unidentified caffeic derivative	288sh, 327	297	179		+	+
21	3-*O*-*p*-Coumaroylquinic acid	310	337	163, 119		+	+
22	3-*O*-caffeoylquinic acid	299sh, 321	353	191	+	+	+
23	Unidentified cumaric derivative	312	281	163		+	+
24	3,4-*O*-dicaffeoylquinic acid	299sh, 327	515	353	+	+	+
25	1,2,3,4-tetra-*O*-galloyl-glucoside	269	787	421	+		
26	1,3,4,6-tetra-*O*-galloyl-glucoside	277	787	421	+		
27	2,3,4,6-tetra-*O*-galloyl-glucoside	278	787	421	+		
28	Apigenin 8-C-glucoside (vitexin)	268, 329	431	311, 341, 284		+	
29	Luteolin 6,8-C-diglucoside	265, 352	609	285		+	
30	Quercetin 3-*O*-rutinoside (rutin)	264, 350	609	301, 187, 111			+
31	Naringenin 7-*O*-glucoside	272, 353	433	271	+		
32	Quercetin 3-*O*-glucoside	255, 355	463	301	+	+	+
33	Quercetin 3-*O*-galactoside	255, 352	463	301	+		+
34	Quercetin 3-*O*-acetyl hexoside	260, 352	505	301			+
35	Myricetin 3-*O*-rhamnoside	281	463	317	+		
36	Apigenin 6-C-glucoside-8-C-arabinoside	269, 329	563	269			+
37	Cratenacin	268, 377	619	413, 293	+	+	+

Abbreviations: B, berries; L, leaves; F, flowers; “+”, compound detected. The table provides a summary of the identified polyphenolic compounds in the extracts from berries (CB1–CB6), leaves (CL1–CL6), and flowers (CF1–CF6).

**Table 2:** Characteristics of the selected *Crataegus* species.

No.	*Crataegus* Species	Samples	Location	
1	*C. monogyna*	CB1; CL1; CF1	Błażowa	49°53′14.55″ N 22°7′16.77″ E
2	*C. rhipidophylla*	CB2; CL2; CF2	Piątkowa	49°53′21.09″ N 22°8′29.93″ E
3	*C. x subsphaericea* (hybrid *C. rhipidophylla* and *C. laevigata*)	CB3; CL3; CF3	Błażowa	49°53′15.24″ N 22°6′46.22″ E
4	*C. laevigata x rhipidophylla x monogyna*	CB4; CL4; CF4	Błażowa	49°53′3.77″ N 22°6′28.68″ E
5	*C. macrocarpa* (hybrid *C. rhipidophylla* and *C. monogyna*)	CB5; CL5; CF5	Błażowa	49°53′4.8″ N 22°6′30.57″ E
6	*C. laevigata*	CB6; CL6; CF6	Piątkowa	49°53′1.89″ N 22°8′59.19″ E

Abbreviations: CB, *Crataegus* berries; CL, *Crataegus* leaves; CF, *Crataegus* flowers.

## Data Availability

Not applicable.

## Supplementary Materials

The following are available online, Figure S1: DMSO effect on the viability of U87MG human glioblastoma cells, Figure S2: Densitometry of PARP1 level, Figure S3: The measure of the level of the phosphorylated (active) form of FAK (p-FAK), Figure S4: Quantification of the level of the phosphorylated (active) form of Akt (p-Akt), Figure S5: UPLC chromatogram of *Crataegus monogyna* berries (CB1), Figure S6: UPLC chromatogram of *Crataegus monogyna* leaves (CL1), Figure S7: UPLC chromatogram of *Crataegus monogyna* flowers (CF1), Table S1: EC50 (µg/mL) values for the examined *Crataegus* extracts on the viability of U87MG human glioblastoma cells, Table S2: Content of polyphenolic compounds in berries, leaves, and flowers of the different *Crataegus* species, Table S3: Extraction yield of particular samples (in %), Table S4: Calibration curve parameters of the method developed for each standard.

## Sample Availability

Samples of the compounds are available from the authors.

## References

[1] Santini A., Novellino E. (2014). Nutraceuticals: Beyond the Diet Before the Drugs. Curr. Bioact. Compd..

[2] Durazzo A., Lucarini M. (2019). Editorial: The State of Science and Innovation of Bioactive Research and Applications, Health, and Diseases. Front. Nutr..

[3] Santini A., Novellino E., Armini V., Ritieni A. (2013). State of the art of Ready-to-Use Therapeutic Food: A tool for nutraceuticals addition to foodstuff. Food Chem..

[4] Durazzo A., Lucarini M., Souto E.B. (2019). Polyphenols: A concise overview on the chemistry, occurrence, and human health. Phytother. Res..

[5] Santini A., Tenore G.C., Novellino E. (2017). Nutraceuticals: A paradigm of proactive medicine. Eur. J. Pharm. Sci..

[6] Rastogi S., Pandey M.M., Rawat A.K.S. (2016). Traditional herbs: A remedy for cardiovascular disorders. Phytomedicine.

[7] Dong Y., Liao J., Yao K., Jiang W., Wang J. (2017). Application of Traditional Chinese Medicine in Treatment of Atrial Fibrillation. Evid. Based Complementary Altern. Med..

[8] Tadić V.M., Dobrić S., Marković G.M., Dordević S.M., Arsić I.A., Menković N.R., Stević T. (2008). Anti-inflammatory, Gastroprotective, Free-Radical-Scavenging, and Antimicrobial Activities of Hawthorn Berries Ethanol Extract. J. Agric. Food Chem..

[9] Strugała P., Gładkowski W., Kucharska A.Z., Sokół-Łętowska A., Gabrielska J. (2016). Antioxidant activity and anti-inflammatory effect of fruit extracts from blackcurrant, chokeberry, hawthorn, and rosehip, and their mixture with linseed oil on a model lipid membrane. Eur. J. Lipid Sci. Technol..

[10] Liu H., Liu J., Lv Z., Yang W., Zhang C., Chen D., Jiao Z. (2019). Effect of dehydration techniques on bioactive compounds in hawthorn slices and their correlations with antioxidant properties. J. Food Sci. Technol..

[11] Lou X., Yuan B., Wang L., Xu H., Hanna M., Yuan L. (2020). Evaluation of physicochemical characteristics, nutritional composition and antioxidant capacity of Chinese organic hawthorn berry (*Crataegus pinnatifida*). Int. J. Food Sci. Technol..

[12] Hwang E., Park S.Y., Yin C.S., Kim H.T., Kim Y.M., Yi T.H. (2017). Antiaging effects of the mixture of *Panax ginseng* and *Crataegus pinnatifida* in human dermal fibroblasts and healthy human skin. J. Ginseng Res..

[13] Lim D.W., Han T., Jung J., Song Y., Um M.Y., Yoon M., Kim Y.T., Cho S., Kim I.H., Han D. (2018). Chlorogenic Acid from Hawthorn Berry (*Crataegus pinnatifida* Fruit) Prevents Stress Hormone-Induced Depressive Behavior, through Monoamine Oxidase B-Reactive Oxygen Species Signaling in Hippocampal Astrocytes of Mice. Mol. Nutr. Food Res..

[14] Bisignano C., Furneri P.M., Mandalari G. (2016). In Vitro Efficacy of *Crataegus oxycantha* L. (Hawthorn) and Its Major Components against ATCC and Clinical Strains of *Ureaplasma urealyticum*. Adv. Microbiol..

[15] Kalantari H., Hemmati A.A., Goudarzi M., Foruozandeh H., Kalantar M., Aghel N., Aslani M.K., Ehsan T.S. (2016). Healing Effect of Hawthorn (*Crataegus pontica* C. Koch) Leaf Extract in Dermal Toxicity Induced by T-2 Toxin in Rabbit. Jundishapur J. Nat. Pharm. Prod..

[16] Niu Z., Yan M., Zhao X., Jin H., Gong Y. (2020). Effect of hawthorn seed extract on the gastrointestinal function of rats with diabetic gastroparesis. S. Afr. J. Bot..

[17] Zhu R.G., Sun Y.D., Hou Y.T., Fan J.G., Chen G., Li T.P. (2017). Pectin penta-oligogalacturonide reduces cholesterol accumulation by promoting bile acid biosynthesis and excretion in high-cholesterol-fed mice. Chem. Biol. Interact..

[18] Edwards J.E., Brown P.N., Talent N., Dickinson T.A., Shipley P.R. (2012). A review of the chemistry of the genus *Crataegus*. Phytochemistry.

[19] Venskutonis P.R. (2018). Phytochemical composition and bioactivities of hawthorn (*Crataegus* spp.): Review of recent research advances. J. Food Bioact..

[20] Guo R., Lin B., Shang X.Y., Zhou L., Yao G.D., Huang X.X., Song S.J. (2018). Phenylpropanoids from the fruit of *Crataegus pinnatifida* exhibit cytotoxicity on hepatic carcinoma cells through apoptosis induction. Fitoterapia.

[21] Mustapha N., Bzéouich I.M., Ghedira K., Hennebelle T., Chekir-Ghedira L. (2015). Compounds isolated from the aerial part of *Crataegus azarolus* inhibit growth of B16F10 melanoma cells and exert a potent inhibition of the melanin synthesis. Biomed. Pharmacother..

[22] Aissani N., Albouchi F., Sebai H. (2020). Anticancer Effect in Human Glioblastoma and Antioxidant Activity of *Petroselinum crispum L.* Methanol Extract. Nutr. Cancer..

[23] Amador S., Nieto-Camacho A., Ramírez-Apan T., Martínez M., Maldonado E. (2020). Cytotoxic, anti-inflammatory, and α-glucosidase inhibitory effects of flavonoids from *Lippia graveolens* (Mexican oregano). Med. Chem. Res..

[24] Karatsai O., Stasyk O., Redowicz M.J. (2020). Effects of Arginine and Its Deprivation on Human Glioblastoma Physiology and Signaling. Adv. Exp. Med. Biol..

[25] Lyons S.M., Alizadeh E., Mannheimer J., Schuamberg K., Caste J., Schroder B., Turk P., Thamm D., Prasad A. (2016). Changes in cell shape are correlated with metastatic potential in murine and human osteosarcomas. Biol. Open..

[26] Saraste A., Pulkki K. (2000). Morphologic and biochemical hallmarks of apoptosis. Cardiovasc. Res..

[27] Zhao X., Guan J.L. (2011). Focal adhesion kinase and Its signaling pathways in cell migration and angiogenesis. Adv. Drug Deliv. Rev..

[28] Zhang J., Hochwald S.N. (2014). The role of FAK in tumor metabolism and therapy. Pharmacol. Ther..

[29] Fayard E., Tintignac L.A., Baudry A., Hemmings B.A. (2005). Protein kinase B/Akt at a glance. J. Cell Sci..

[30] Nunes M.A., Rodrigues F., Alves R.C., Oliveira M.B.P. (2017). Herbal products containing *Hibiscus sabdariffa* L., *Crataegus* spp., and *Panax* spp.: Labeling and safety concerns. Food Res. Int..

[31] Wallace T.C., Giusti M.M. (2015). Anthocyanins. Adv. Nutr..

[32] Vogiatzoglou A., Mulligan A.A., Luben R.N., Lentjes M.A., Heiss C., Kelm M., Merx M.W., Spencer J.P., Schroeter H., Kuhnle G.G. (2014). Assessment of the dietary intake of total flavan-3-ols, monomeric flavan-3-ols, proanthocyanidins and theaflavins in the European Union. Br. J. Nutr..

[33] Aron P.M., Kennedy J.A. (2008). Flavan-3-ols: Nature, occurrence and biological activity. Mol. Nutr. Food Res..

[34] Gu L., Kelm M.A., Hammerstone J.F., Zhang Z., Beecher G., Holden J., Haytowitz D., Prior R.L. (2003). Liquid chromatographic/electrospray ionization mass spectrometric studies of proanthocyanidins in foods. J. Mass Spectrom..

[35] Chen S.D., Lu C.J., Zhao R.Z. (2014). Qualitative and Quantitative Analysis of Rhizoma Smilacis glabrae by Ultra High Performance Liquid Chromatography Coupled with LTQ OrbitrapXL Hybrid Mass Spectrometry. Molecules.

[36] Barbehenn R.V., Constabel C. (2011). Tannins in plant–herbivore interactions. Phytochemistry.

[37] Zafrilla P., Ferreres F., Tomás-Barberán F.A. (2001). Effect of Processing and Storage on the Antioxidant Ellagic Acid Derivatives and Flavonoids of Red Raspberry (*Rubus idaeus*) Jams. J. Agric. Food Chem..

[38] Singh A., Bajpai V., Kumar S., Sharma K.R., Kumar B. (2016). Profiling of Gallic and Ellagic Acid Derivatives in Different Plant Parts of *Terminalia Arjuna* by HPLC-ESI-QTOF-MS/MS. Nat. Prod. Commun..

[39] Xu M., Liu P., Jia X., Zhai M., Zhou S., Wu B., Guo Z. (2020). Metabolic profiling revealed the organ-specific distribution differences of tannins and flavonols in pecan. Food Sci. Nutr..

[40] Sánchez-Rabaneda F., Jáuregui O., Casals I., Andrés-Lacueva C., Izquierdo-Pulido M., Lamuela-Raventós R.M. (2003). Liquid chromatographic/electrospray ionization tandem mass spectrometric study of the phenolic composition of cocoa (*Theobroma cacao*): LC/ESI-MS/MS identification of flavonoids in cocoa. J. Mass Spectrom..

[41] Sasmita A.O., Wong Y.P., Ling A.P.K. (2018). Biomarkers and therapeutic advances in glioblastoma multiforme. Asia Pac. J. Clin. Oncol..

[42] Alexander B.M., Cloughesy T.F. (2017). Adult Glioblastoma. J. Clin. Oncol..

[43] Mraihi F., Fadhil H., Trabelsi-Ayadi M., Chérif J.K. (2015). Chemical characterization by HPLC-DAD-ESI/MS of flavonoids from hawthorn fruits and their inhibition of human tumor growth. J. New Sci..

[44] Ganie S.A., Dar T.A., Zargar S., Bhat A.H., Dar K.B., Masood A., Zargar M.A. (2016). *Crataegus songarica* methanolic extract accelerates enzymatic status in kidney and heart tissue damage in albino rats and Its in vitro cytotoxic activity. Pharm. Biol..

[45] Belkhir M., Dhaouadi K., Rosa A., Atzeri A., Nieddu M., Ignazio C., Tuberoso G., Rescigno A., Amri M., Fattouch S. (2016). Protective effects of azarole polyphenolic extracts against oxidative damage using in vitro biomolecular and cellular models. Ind. Crops Prod..

[46] Bura F.T., Firuzja R.A., Nemati F. (2016). Cytotoxic effect of the flower and leaf bud extract of *Crataegus microphylla* C. Koch on Hela cell line. IIOAB J..

[47] Lou X., Xu H., Hanna M., Yuan L. (2020). Identification and quantification of free, esterified, glycosylated and insoluble-bound phenolic compounds in hawthorn berry fruit (*Crataegus pinnatifida*) and antioxidant activity evaluation. LWT.

[48] Coimbra A.T., Luís Â.F.S., Batista M.T., Ferreira S.M.P., Duarte A.P.C. (2020). Phytochemical Characterization, Bioactivities Evaluation and Synergistic Effect of *Arbutus unedo* and *Crataegus monogyna* Extracts with Amphotericin B. Curr. Microbiol..

[49] Issaadi O., Fibiani M., Picchi V., Scalzo R.L., Madani K. (2020). Phenolic composition and antioxidant capacity of hawthorn (*Crataegus oxyacantha* L.) flowers and fruits grown in Algeria. J. Complement Integr. Med..

[50] Sánchez-Velázquez O.A., Cortés-Rodríguez M., Milán-Carrillo J., Montes-Avila J., Robles-Banuelos B., Angel A.S., Cuevas-Rodriguez E.O., Rangel-Lopes E. (2020). Anti-oxidant and anti-proliferative effect of anthocyanin enriched fractions from two Mexican wild blackberries (*Rubus* spp.) on HepG2 and glioma cell lines. J. Berry Res..

[51] Provenzano F., Sánchez J.L., Rao E., Santonocito R., Ditta L.A., Linares I.B., Passantino R., Campisi P., Dia M.G., Costa M.A. (2019). Water Extract of *Cryphaea heteromalla* (Hedw.) D. Mohr Bryophyte as a Natural Powerful Source of Biologically Active Compounds. Int. J. Mol. Sci..

[52] Ooi K.L., Muhammad T.S.T., Tan M.L., Sulaiman S.F. (2011). Cytotoxic, apoptotic and anti-α-glucosidase activities of 3,4-di-*O*-caffeoyl quinic acid, an antioxidant isolated from the polyphenolic-rich extract of *Elephantopus mollis* Kunth. J. Ethnopharmacol..

[53] Murad L.D., Soares N.P., Brand C., Monteiro M.C., Teodoro A.J. (2015). Effects of Caffeic and 5-Caffeoylquinic Acids on Cell Viability and Cellular Uptake in Human Colon Adenocarcinoma Cells. Nutr. Cancer..

[54] Kim D.O., Lee C.Y. (2004). Comprehensive Study on Vitamin C Equivalent Antioxidant Capacity (VCEAC) of Various Polyphenolics in Scavenging a Free Radical and its Structural Relationship. Crit. Rev. Food Sci. Nutr..

[55] Soica C., Oprean C., Borcan F., Danciu C., Trandafirescu C., Coricovac D., Crainiceanu Z., Dehelean C.A., Munteanu M. (2014). The Synergistic Biologic Activity of Oleanolic and Ursolic Acids in Complex with Hydroxypropyl-γ-Cyclodextrin. Molecules.

[56] Karatsai O., Shliaha P., Jensen O.N., Stasyk O., Rędowicz M.J. (2020). Combinatory Treatment of Canavanine and Arginine Deprivation Efficiently Targets Human Glioblastoma Cells via Pleiotropic Mechanisms. Cells.

