# Supplementary material for: Polyphenolic Compounds of Crataegus Berry, Leaf, and Flower Extracts Affect Viability and Invasive Potential of Human Glioblastoma Cells
Source: Molecules. 2021 May 1;26(9):2656. doi: 10.3390/molecules26092656 (PMC8124274; doi:10.3390/molecules26092656)
Supplement: Supplementary file 1 [file molecules-26-02656-s001.zip › molecules-1185795-supplementary.pdf]

molecules

Supplementary material

# Polyphenolic Compounds of Crataegus Berry, Leaf and Flower Extracts Affect Viability and Invasive Potential of Human Glioblastoma Cells

**Natalia Żurek [1,a], Olena Karatsai [2,a], Maria Jolanta Rędowicz [1,2,*,b] and Ireneusz Kapusta [1,b]**

[1] Institute of Food Technology and Nutrition, University of Rzeszow, 4 Zelwerowicza St., 35-601 Rzeszow, Poland; nzurek@ur.edu.pl, ikapusta@ur.edu.pl, j.redowicz@nencki.edu.pl
[2] Nencki Institute of Experimental Biology, Polish Academy of Sciences, 3 Pasteur St., 02-093 Warsaw, Poland; o.karatsai@nencki.edu.pl, j.redowicz@nencki.edu.pl
* Correspondence: ikapusta@ur.edu.pl; Tel.: +48-17-785-5238
[a] These authors contributed equally to this article
[b] These authors share senior authorship

Content:

**Supplementary Figure S1:** DMSO effect on the viability of U87MG human glioblastoma cells.
**Supplementary Figure S2:** Densitometry of PARP1 level.
**Supplementary Figure S3:** The measure of the level of the phosphorylated (active) form of FAK (p-FAK).
**Supplementary Figure S4:** Quantification of the level of the phosphorylated (active) form of Akt (p-Akt).
**Supplementary Figure S5:** UPLC chromatogram of *Crataegus monogyna* berries (CB1).
**Supplementary Figure S6:** UPLC chromatogram of *Crataegus monogyna* leaves (CL1).
**Supplementary Figure S7:** UPLC chromatogram of *Crataegus monogyna* flowers (CF1).
**Supplementary Table S1:** EC50 (μg/mL) values for the examined *Crataegus* extracts on the viability of U87MG human glioblastoma cells.
**Supplementary Table S2:** Content of polyphenolic compounds in berries, leaves and flowers of the different *Crataegus* species.

**Supplementary Table S3:** Extraction yield of particular samples (in %)
**Supplementary Table S4:** Calibration curve parameters of the method developed for each standard

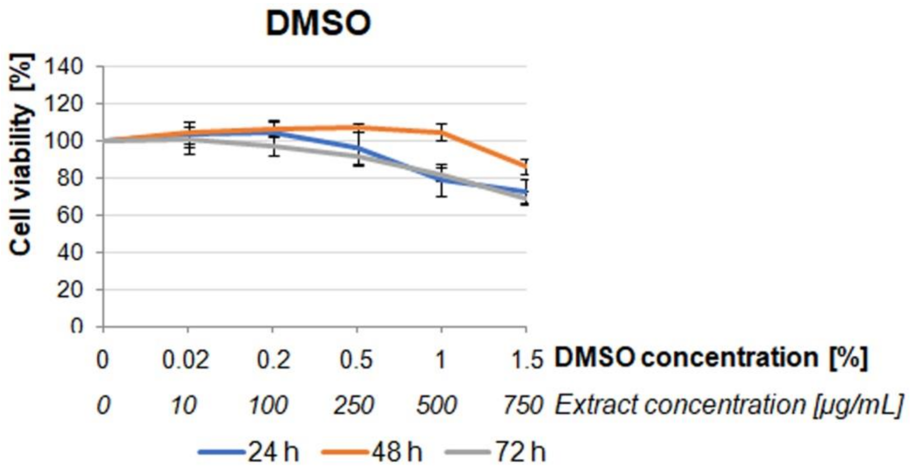


**Figure S1.** DMSO effect on the viability of U87MG human glioblastoma cells. Cells were treated up to 72 hours with different concentrations of DMSO (0.02-1.5%) deppending on the flower extracts concentration (see lower raw). The viability

was assayed by the MTS test. The number of viable control (non-treated) cells of each time point served as 100%. Graphs represent mean values ± SD from three independent experiments.

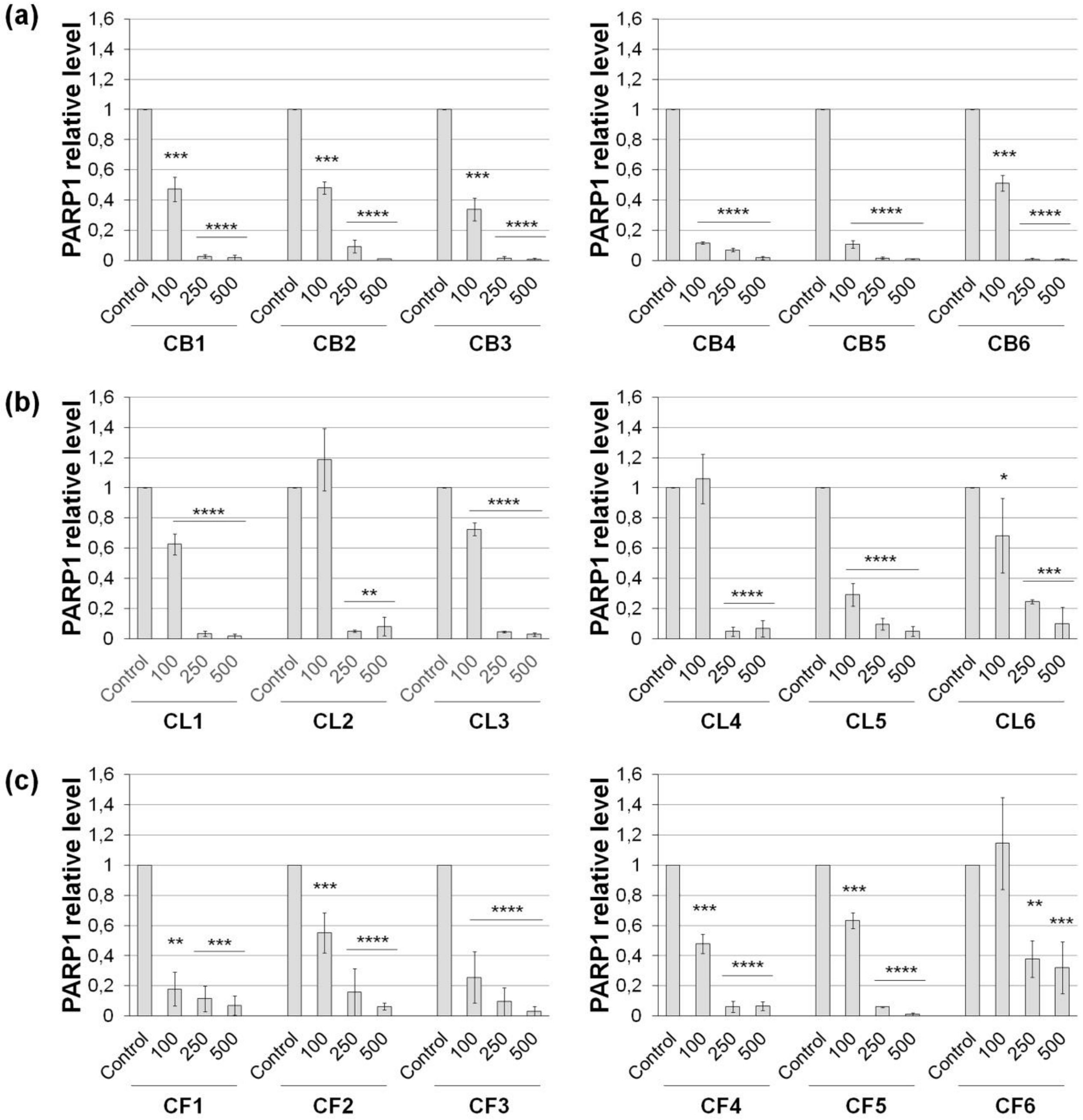


**Figure S2.** Densitometric analysis of the PARP1 level. Cells were incubated with *Crataegus* berry (**a**), leaf (**b**) and flower (**c**) extracts in 100, 250, and 500 µg/mL concentrations for 48 h. Non-treated cells served as a control. Graphs represent mean value ± SD. * $p < 0.05$, ** $p < 0.01$, *** $p < 0.001$, **** $p < 0.0001$ relative to control.

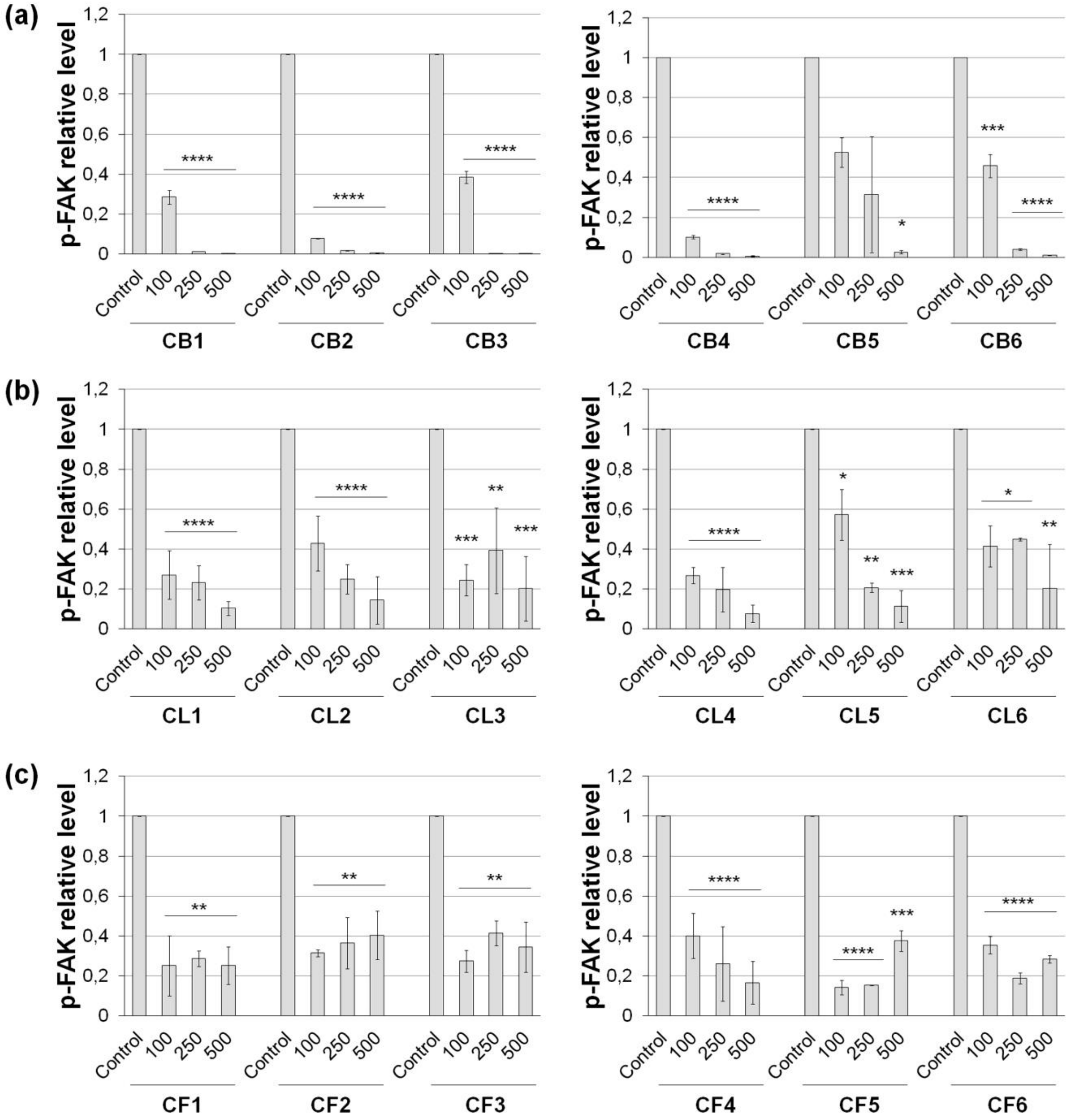


**Figure S3.** Densitometric analysis of the level of phosphorylated (active) form of FAK (p-FAK). Cells were incubated with *Crataegus* berry (**a**), leaf (**b**) and flower (**c**) extracts in 100, 250, and 500 µg/mL concentrations for 48 h. Non-treated cells served as a control. Graphs represent mean value ± SD. * $p < 0.05$, ** $p < 0.01$, *** $p < 0.001$, **** $p < 0.0001$ relative to control.

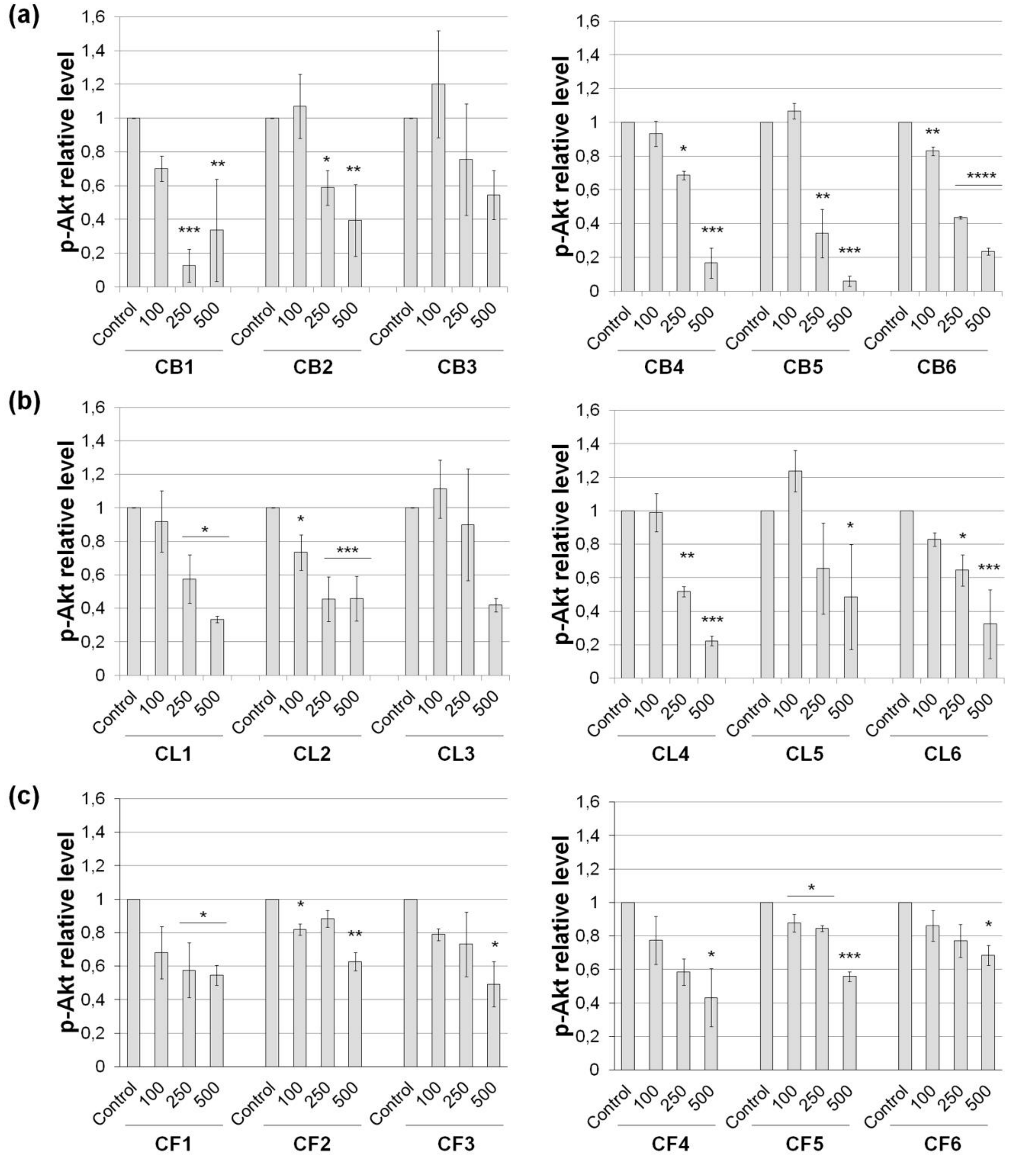


**Figure S4.** Densitometric analysis of the level of phosphorylated (active) form of Akt (p-Akt). Cells were incubated with *Crataegus* berry (**a**), leaf (**b**) and flower (**c**) extracts in 100, 250, and 500 µg/mL concentrations for 48 h. Non-treated cells served as a control. Graphs represent mean value ± SD. * $p < 0.05$, ** $p < 0.01$, *** $p < 0.001$, **** $p < 0.0001$ relative to control.

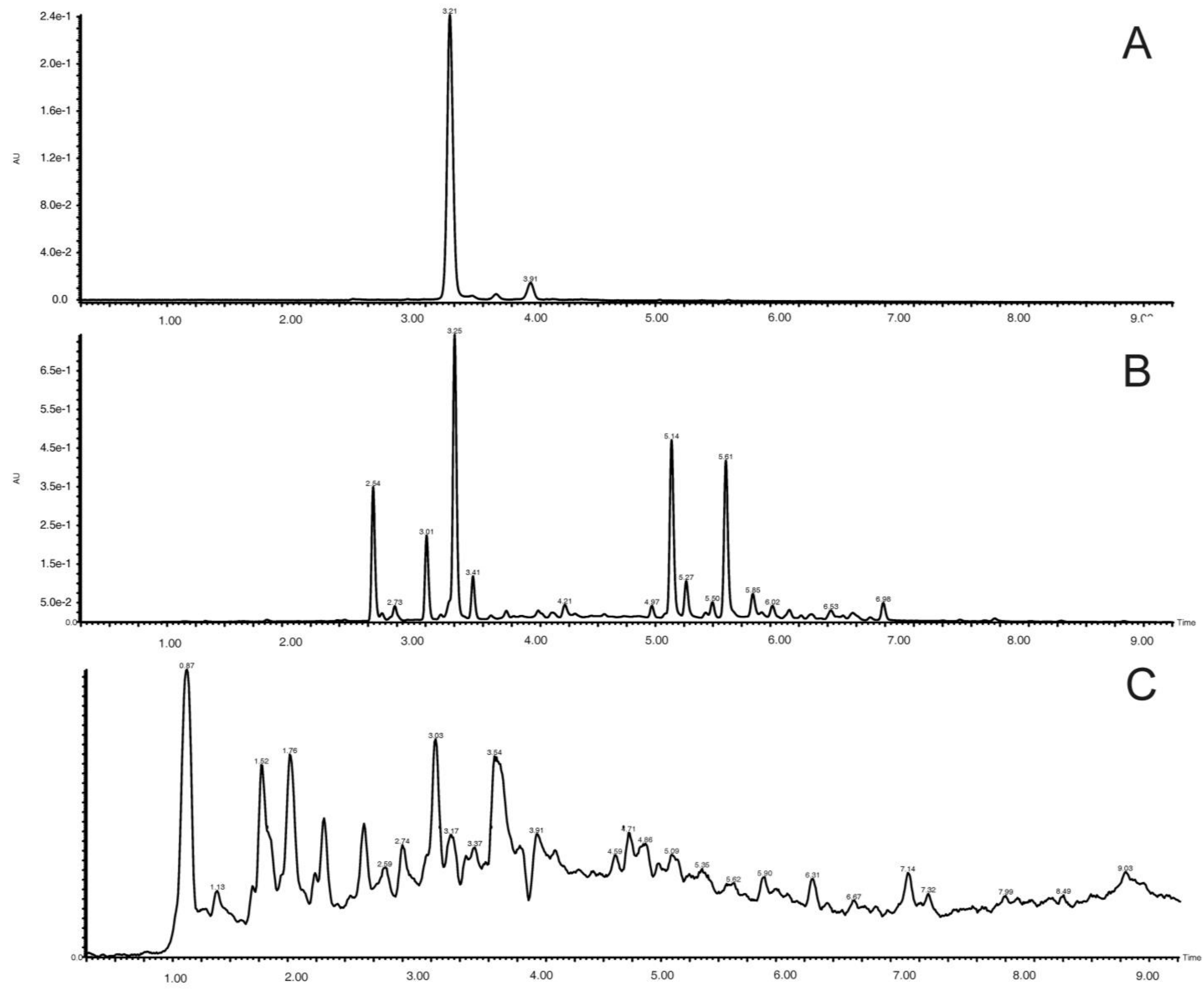


**Figure S5.** UPLC chromatogram of *Crataegus monogyna* berries (CB1). A, PDA chromatogram extracted at 520 nm for anthocyanins visualization, B, PDA chromatogram extracted at 350 nm for other phenolics, C – total ion current.

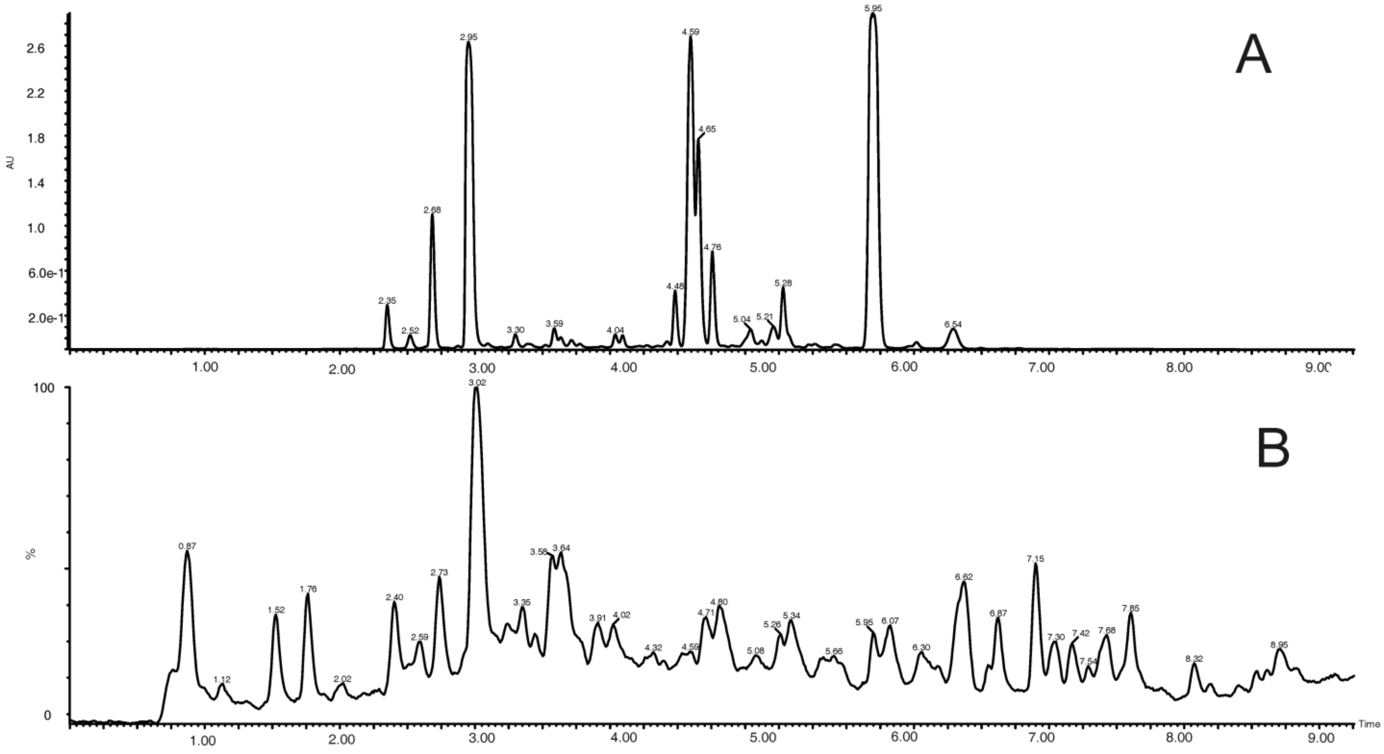


**Figure S6.** UPLC chromatogram of *Crataegus monogyna* leaves (CL1). A, PDA chromatogram extracted at 350 nm, B, total ion current.

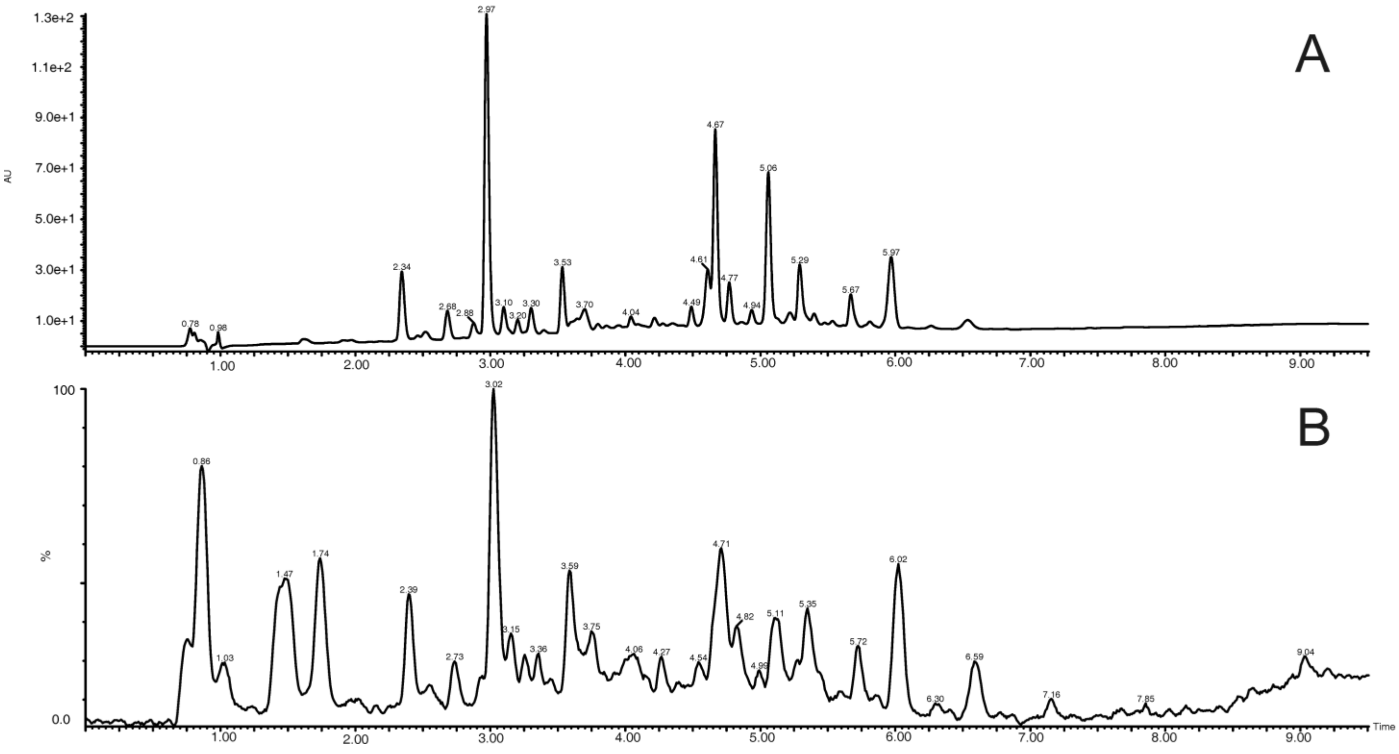


**Figure S7.** UPLC chromatogram of *Crataegus monogyna* flowers (CF1). A, PDA chromatogram extracted at 350 nm, B, total ion current.

**Table S1.** EC50 (μg/mL) values for the examined *Crataegus* extracts on the viability of U87MG human glioblastoma cells.

| Samples | EC50, μg/mL | | |
|---|---|---|---|
| | Time, h | | |
| | 24 | 48 | 72 |
| CB1 | 200.26 ± 9.52 | 189.33 ± 4.87 | 181.87 ± 6.03 |
| CB2 | 198.55 ± 6.34 | 198.83 ± 4.32 | 183.89 ± 3.02 |
| CB3 | 168.65 ± 11.71 | 189.00 ± 15.09 | 209.49 ± 10.41 |
| CB4 | 180.88 ± 8.57 | 173.70 ± 5.50 | 180.44 ± 8.31 |
| CB5 | 227.88 ± 9.12 | 256.79 ± 9.11 | 335.42 ± 7.00 |
| CB6 | 356.82 ± 5.90 | 344.35 ± 7.61 | 395.09 ± 6.09 |
| | | | |
| CL1 | 218.79 ± 7.77 | 233.44 ± 2.70 | 214.41 ± 3.43 |
| CL2 | 450.96 ± 10.78 | 451.59 ± 15.02 | 403.36 ± 22.66 |
| CL3 | 263.12 ± 24.97 | 338.37 ± 6.58 | 310.73 ± 9.36 |
| CL4 | 185.84 ± 5.89 | 116.51 ± 22.21 | 184.27 ± 1.22 |
| CL5 | 174.01 ± 19.09 | 170.08 ± 2.69 | 207.07 ± 10.58 |
| CL6 | 236.67 ± 5.58 | 338.26 ± 11.88 | 379.43 ± 17.37 |
| | | | |
| CF1 | 343.02 ± 46.48 | 426.44 ± 12.95 | 460.29 ± 10.53 |
| CF2 | 462.38 ± 8.09 | 553.58 ± 13.47 | 551.79 ± 25.92 |
| CF3 | 429.91 ± 13.24 | 569.93 ± 17.74 | 559.15 ± 15.34 |
| CF4 | 329.95 ± 26.88 | 391.24 ± 8.53 | 440.99 ± 13.47 |
| CF5 | 636.48 ± 20.69 | 565.96 ± 17.66 | 523.63 ± 28.60 |
| CF6 | 629.78 ± 11.09 | 543.19 ± 30.67 | 440.29 ± 13.59 |

Values are expressed as mean ± SD from three independent experiments. Abbreviations: CB, *Crataegus* berry; CL, *Crataegus* leaf; CF, *Crataegus* flower extracts.

**Table S2.** Content of polyphenolic compounds in berries, leaves and flowers of the different *Crataegus* species.

| | Compounds | | Species of *Crataegus* | | | | | |
|---|---|---|---|---|---|---|---|---|
| | | | 1 | 2 | 3 | 4 | 5 | 6 |
| Berries | *Anthocyanins* | | | | | | | |
| | 1 | Cyanidin 3-*O*-glucoside | 7.37 ± 0.07d | 6.42 ± 0.01b | 5.75 ± 0.01a | 11.21 ± 0.05e | 12.79 ± 0.27f | 6.96 ± 0.02c |
| | 2 | Pelargonidin 3-*O*-rutinoside | 0.15 ± 0.02b | 0.12 ± 0.01a | 0.11 ± 0.01a | 0.22 ± 0.01c | 0.22 ± 0.00c | 0.16 ± 0.00b |
| | 3 | Cyanidin 3-*O*-arabinoside | 0.13 ± 0.00b | 0.14 ± 0.01b | 0.11 ± 0.00a | 0.24 ± 0.01c | 0.25 ± 0.01c | 0.26 ± 0.00d |
| | 4 | Peonidin 3-*O*-glucoside | 0.41 ± 0.00c | 0.42 ± 0.02c | 0.29 ± 0.00b | 0.25 ± 0.00ab | 0.25 ± 0.04ab | 0.21 ± 0.00a |
| | | Sum | 8.07 ± 0.09d | 7.09 ± 0.13b | 6.26 ± 0.02a | 11.91 ± 0.07e | 13.51 ± 0.32f | 7.59 ± 0.03c |
| | *Flavan-3-ols* | | | | | | | |
| | 5 | Procyanidin trimer | 1439.52 ± 21.62a | 1763.48 ± 18.20c | 1599.89 ± 4.70b | 2000.45 ± 21.32de | 2048.89 ± 10.93e | 1877.19 ± 1.29cd |
| | 6 | Procyanidin dimer | 1717.54 ± 43.39a | 2294.92 ± 13.81c | 2009.59 ± 19.49b | 2578.00 ± 17.93d | 2762.86 ± 7.34e | 2636.03 ± 20.84de |

| | | | | | | | |
|---|---|---|---|---|---|---|---|
| 7 | (+)-Catechin | 213.06 ± 30.29c | 88.35 ± 5.30a | 61.40 ± 1.93a | 1073.04 ± 40.66d | 1451.14 ± 1.46e | 153.14 ± 7.14b |
| 8 | (-)-Epicatechin | 1399.75 ± 51.77a | 1793.75 ± 5.68b | 1419.88 ± 16.08a | 1800.44 ± 10.24b | 1839.19 ± 17.83b | 2092.24 ± 13.66c |
| 9 | Procyanidin tetramer | 114.36 ± 1.41a | 162.62 ± 5.71b | 145.14 ± 6.67b | 215.74 ± 7.61c | 213.73 ± 4.76c | 140.16 ± 2.94ab |
| 10 | Cinchonain | 29.10 ± 0.66b | 61.47 ± 7.29c | 0.33 ± 0.38a | 4.27 ± 0.23a | 4.49 ± 0.08a | 1.32 ± 0.40a |
| | Sum | 4913.36 ± 149.14a | 6164.60 ± 56.00c | 5236.21 ± 49.26b | 7671.94 ± 97.99e | 8320.29 ± 42.41f | 6900.07 ± 57.91d |
| | *Hydrolyzable Tannins* | | | | | | |
| 11 | Ellagic acid pentoside | 52.31 ± 4.22ab | 78.27 ± 4.54b | 540.44 ± 19.81c | 25.18 ± 1.46a | 56.50 ± 2.75ab | 679.17 ± 23.19d |
| 12 | Punicalin isomer I | 293.34 ± 13.08a | 367.42 ± 11.62b | 357.38 ± 3.79b | 408.15 ± 18.88c | 426.93 ± 0.08c | 406.85 ± 21.33c |
| 13 | Punicalin isomer II | 39.31 ± 4.54a | 22.52 ± 1.75a | 60.82 ± 4.05a | 42.49 ± 2.62a | 113.56 ± 0.65b | 57.17 ± 38.78a |
| 14 | 2-*O*-galloylpunicalin | 52.73 ± 2.80a | 59.47 ± 8.50a | 86.05 ± 3.72b | 135.17 ± 8.86c | 136.76 ± 0.85c | 56.38 ± 16.98a |
| 15 | Eucalbanin A | 13.34 ± 0.70a | 19.75 ± 0.06b | 21.81 ± 1.44b | 22.41 ± 1.06b | 15.37 ± 0.80a | 22.65 ± 2.95b |
| | Sum | 451.04 ± 25.35a | 547.43 ± 26.47b | 1066.50 ± 32.82e | 633.41 ± 32.88c | 749.13 ± 5.14d | 1222.22 ± 103.25f |

| | | | | | | |
|---|---|---|---|---|---|---|
| *Phenolic acids* | | | | | | |
| 16 | Quinic acid | 25.91 ± 1.16a | 28.18 ± 2.38a | 31.50 ± 6.13a | 28.43 ± 2.95a | [illegible] |
| 17 | Coumaroylquinic acid | 118.21 ± 1.18a | 124.46 ± 5.87a | 113.45 ± 2.33a | 194.09 ± 3.42b | [illegible] |
| 18 | Protocatechuic acid glucoside | 54.25 ± 1.05c | 181.48 ± 9.46f | 39.29 ± 4.06b | 100.26 ± 3.69d | [illegible] |
| 19 | 4-*O*-caffeoylquinic acid | 737.15 ± 23.08d | 457.81 ± 4.47c | 1043.00 ± 2.21e | 1955.17 ± 7.62f | [illegible] |
| 22 | 3-*O*-caffeoylquinic acid | 745.73 ± 38.11c | 867.99 ± 2.97d | 319.66 ± 1.09a | 1200.17 ± 9.13e | [illegible] |
| 24 | 3,4-*O*-dicaffeoylquinic acid | 101.86 ± 8.38d | 42.11 ± 1.47c | 13.35 ± 0.29a | 23.81 ± 5.83ab | [illegible] |
| | Sum | 1783.12 ± 72.95d | 1702.03 ± 26.62c | 1560.25 ± 16.12b | 3501.93 ± 32.64e | [illegible] |
| *Flavonols* | | | | | | |
| 25 | 1,2,3,4-tetra-*O*-galloyl-glucoside | 8.68 ± 0.48c | 5.99 ± 0.38b | 9.72 ± 0.19c | 6.00 ± 0.53b | [illegible] |
| 26 | 1,3,4,6-tetra-*O*-galloyl-glucoside | 18.70 ± 1.70b | 23.52 ± 3.27bc | 9.58 ± 3.50a | 34.82 ± 2.46d | [illegible] |
| 27 | 2,3,4,6-tetra-*O*-galloyl-glucoside | 87.88 ± 2.88b | 95.78 ± 0.13b | 59.04 ± 2.78a | 144.69 ± 6.69d | [illegible] |
| 31 | Naringenin 7-*O*-glucoside | 34.97 ± 1.02b | 8.39 ± 0.63a | 35.77 ± 0.82b | 29.42 ± 4.71b | [illegible] |
| 32 | Quercetin 3-*O*-glucoside | 1062.82 ± 51.49c | 662.74 ± 20.55a | 1322.16 ± 3.80d | 1409.36 ± 23.48e | [illegible] |
| 33 | Quercetin 3-*O*-galactoside | 345.65 ± 18.51d | 191.83 ± 5.26b | 455.82 ± 6.39e | 297.54 ± 6.92c | [illegible] |
| 35 | Myricetin 3-*O*-rhamnoside | 114.94 ± 2.48c | 523.56 ± 4.34e | 56.89 ± 0.71a | 117.30 ± 4.49c | [illegible] |
| 37 | Cratenacin | 78.92 ± 6.67c | 3.27 ± 0.79a | nd | 72.67 ± 2.95c | [illegible] |
| | Sum | 1752.57 ± 85.23c | 1515.09 ± 35.35b | 1948.98 ± 18.18d | 2111.81 ± 52.23e | [illegible] |
| | **Total (mg/100 g d.b.)** | **8980.77 ± 332.76**a | **10000.07 ± 144.57**b | **9874.49 ± 116.40**b | **14038.21 ± 215.81**e | [illegible] |
| *Flavan-3-ols* | | | | | | |
| 5 | Procyanidin trimer | 78.78 ± 1.34c | 57.75 ± 4.24ab | 44.74 ± 12.91ab | 62.93 ± 1.63bc | [illegible] |

| | | | | | | | |
|---|---|---|---|---|---|---|---|
| | 6 | Procyanidin dimer | 904.13 ± 4.15d | 759.88 ± 1.32c | 1475.91 ± 1.03f | 719.50 ± 1.04b | [illegible] |
| | 7 | (+)-Catechin | 167.68 ± 7.26c | 168.51 ± 5.71c | 93.74 ± 9.88a | 89.44 ± 1.00a | [illegible] |
| | | Sum | 1150.59 ± 12.75e | 986.14 ± 11.28d | 1614.39 ± 23.82f | 871.88 ± 3.66b | [illegible] |
| | *Hydrolyzable Tannins* | | | | | | |
| | 12 | Punicalin isomer I | 75.05 ± 0.11d | 51.03 ± 4.82b | 48.16 ± 0.04b | 70.20 ± 0.56d | [illegible] |
| | | Sum | 75.05 ± 0.11d | 51.03 ± 4.82b | 48.16 ± 0.04b | 70.20 ± 0.56d | [illegible] |
| | *Phenolic acids* | | | | | | |
| | 20 | Unidentified caffeic derivative | 441.64 ± 7.42d | 264.98 ± 2.83b | 188.07 ± 3.82a | 180.78 ± 3.06a | [illegible] |
| | 21 | 3-*O*-*p*-coumaroylquinic acid | 357.04 ± 5.87b | 179.17 ± 9.79a | 197.64 ± 33.50a | 223.34 ± 3.79a | [illegible] |
| | 22 | 3-*O*-caffeoylquinic acid | 1463.94 ± 6.23e | 1247.22 ± 8.88d | 1151.22 ± 12.52c | 1026.81 ± 6.46b | [illegible] |
| | 23 | Unidentified cumaric derivative | 131.93 ± 1.88f | 103.63 ± 0.37e | 79.37 ± 0.50b | 25.35 ± 0.53a | [illegible] |
| | 24 | 3,4-*O*-dicaffeoylquinic acid | 197.09 ± 7.27e | 127.37 ± 12.40c | 53.98 ± 0.17a | 91.05 ± 9.16b | [illegible] |
| | | Sum | 2591.63 ± 28.66d | 1922.37 ± 34.27c | 1670.28 ± 50.51b | 1547.32 ± 23.01a | [illegible] |
| | *Flavonols* | | | | | | |
| | 28 | Apigenin 8-C-glucoside (vitexin) | 48.57 ± 3.57c | 38.20 ± 3.18b | 17.42 ± 2.64a | 35.10 ± 4.36b | [illegible] |
| | 29 | Luteolin 6,8-C-diglucoside | 50.81 ± 0.18bc | 79.51 ± 1.75d | 94.14 ± 0.03e | 52.44 ± 3.30c | [illegible] |
| | 32 | Quercetin 3-*O*-glucoside | 674.60 ± 0.84f | 479.28 ± 3.48c | 413.87 ± 1.93a | 598.10 ± 2.05e | [illegible] |
| | 37 | Cratenacin | 1672.30 ± 2.03f | 1315.66 ± 1.90b | 104.89 ± 7.34a | 1659.41 ± 8.81e | [illegible] |
| | | Sum | 2446.28 ± 6.63f | 1912.65 ± 10.31c | 630.32 ± 11.95a | 2345.05 ± 18.52e | [illegible] |
| | | **Total (mg/100 g d.b.)** | **6263.56 ± 48.04**d | **4872.18 ± 55.86**c | **3963.15 ± 86.27**a | **4834.46 ± 45.19**c | [illegible] |
| **Flowers** | *Flavan-3-ols* | | | | | | |
| | 6 | Procyanidin dimer | 192.06 ± 5.27cd | 171.94 ± 1.79a | 187.02 ± 2.85bc | 275.68 ± 2.22e | [illegible] |
| | 7 | (+)-Catechin | 221.65 ± 3.62d | 147.20 ± 5.60b | 160.24 ± 2.39c | 165.86 ± 1.02c | [illegible] |
| | | Sum | 413.71 ± 8.89c | 319.15 ± 7.40a | 347.27 ± 5.25b | 441.54 ± 3.25d | [illegible] |
| | *Phenolic acids* | | | | | | |
| | 20 | Unidentified caffeic derivative | 284.61 ± 7.72d | 68.56 ± 2.63ab | 75.16 ± 1.77b | 78.61 ± 1.68b | [illegible] |
| | 21 | 3-*O*-*p*-coumaroylquinic acid | 551.02 ± 11.27c | 113.29 ± 3.59a | 106.53 ± 0.85a | 294.61 ± 19.07b | [illegible] |
| | 22 | 3-*O*-caffeoylquinic acid | 2588.40 ± 47.41d | 988.45 ± 1.31a | 1077.11 ± 8.51a | 1366.45 ± 6.08b | [illegible] |
| | 23 | Unidentified cumaric derivative | 169.77 ± 2.47e | 51.74 ± 0.01b | 57.89 ± 1.18c | 103.55 ± 2.85d | [illegible] |
| | 24 | 3,4-*O*-di-caffeoylquinic acid | 458.56 ± 16.07e | 140.15 ± 0.30b | 165.82 ± 23.03b | 89.87 ± 0.44a | [illegible] |
| | | Sum | 4052.36 ± 84.94e | 1362.20 ± 7.85a | 1482.51 ± 35.25a | 1933.07 ± 30.13b | [illegible] |
| | *Flavonols* | | | | | | |
| | 30 | Quercetin 3-*O*-rutinoside (rutin) | 35.45 ± 2.00b | 36.66 ± 0.55b | 40.41 ± 0.95c | 56.75 ± 0.35d | [illegible] |
| | 32 | Quercetin 3-*O*-glucoside | 2023.70 ± 11.10d | 496.88 ± 13.09a | 513.48 ± 11.27a | 849.00 ± 28.40b | [illegible] |
| | 33 | Quercetin 3-*O*-galactoside | 284.52 ± 1.30b | 80.44 ± 2.16a | 87.25 ± 2.05a | 107.84 ± 6.87a | [illegible] |
| | 34 | Quercetin 3-*O*-acetyl hexoside | 135.95 ± 7.72b | 398.37 ± 1.08c | 470.60 ± 18.59d | 485.67 ± 21.97d | [illegible] |
| | 36 | Apigenin 6-C-glucoside-8-C-arabinoside | 250.39 ± 7.80d | 77.67 ± 0.24a | 73.23 ± 4.33a | 92.45 ± 5.94b | [illegible] |
| | 37 | Cratenacin | 521.92 ± 8.01f | 257.45 ± 1.02a | 284.17 ± 2.60b | 389.44 ± 6.30c | [illegible] |
| | | Sum | 3251.93 ± 37.93d | 1347.48 ± 18.14a | 1469.14 ± 39.79a | 1981.15 ± 69.85b | [illegible] |

| | | | | | |
|---|---|---|---|---|---|
| **Total (mg/100 g d.b.)** | **7718.00 ± 131.76**f | **3028.82 ± 33.39**a | **3298.92 ± 80.38**b | **4355.76 ± 103.22**c | [illegible] |

Values are given in mg/100 g d.b. (dry basis) and are expressed as mean ± SD. Statistical significance was analyzed with Duncan's test. Values marked with different letters (between species) in the same row indicate statistically significant differences ($p < 0.05$), for more details see Materials and Methods; nd, not detected.

**Table S3.** Extraction yield of particular samples (in %).

| **Samples** | **Extraction yield (%)** | | |
|---|---|---|---|
| | **Berries** | **Leaves** | **Flowers** |
| C1 | 6.27 | 10.05 | 9.34 |
| C2 | 5.73 | 8.34 | 8.98 |
| C3 | 5.25 | 7.69 | 7.43 |
| C4 | 5.69 | 8.61 | 8.40 |
| C5 | 7.40 | 9.38 | 5.83 |
| C6 | 6.32 | 9.47 | 5.93 |

**Table 4.** Calibration curve parameters of the method developed for each standard.

| **No** | **Compound** | **Linearity range [µg/mL]** | **Regresion equation** | **R2** | **LOD [µg/mL]** | **LOQ [µg/mL]** |
|---|---|---|---|---|---|---|
| | | | ***Anthocyanins*** | | | |
| 1 | Cyanidin 3-*O*-glucoside | 25 - 250 | $y = 1.66x10^{-5} – 8.92$ | 0.998 | 0.015 | 0.045 |
| | | | ***Flavan-3-ols*** | | | |
| 2 | (+)catechin | 25 - 250 | $y = 1.4x10^{-5} – 6.36$ | 0.999 | 0.013 | 0.038 |
| | | | ***Hydrolyzable tannins*** | | | |
| 3 | Ellagic acid | 25 - 250 | $y = 2.98x10^{-5} +0.30$ | 0.999 | 0.027 | 0.081 |
| | | | ***Phenolic acids*** | | | |
| 4 | 3-*O*-caffeoylquni c acid | 25 - 250 | $y = 6.16x10^{-5} – 3.40$ | 0.997 | 0.055 | 0.168 |
| 5 | *p*-coumaric acid | 25 - 250 | $y = 8.19x10^{-5} – 0.06$ | 0.999 | 0.074 | 0.22 |
| 6 | Protocatechuic acid | 25 - 250 | $y = 7.8x10^{-5} – 0.04$ | 0.998 | 0.07 | 0.213 |
| | | | ***Flavonols*** | | | |
| 7 | Apigenin 8-C-glucoside | 25 - 250 | $y = 1.33x10^{-5} – 4.02$ | 0.999 | 0.012 | 0.036 |
| 8 | Quercetin 3-*O*-rutinoside | 25 - 250 | $y = 4.85x10^{-5} +0.78$ | 0.999 | 0.048 | 0.132 |
| 9 | Naringenin 7-*O*-glucoside | 25 - 250 | $y = 4.78x10^{-5} – 7.86$ | 0.999 | 0.043 | 0.13 |
| 10 | Luteolin 7-*O*-glucoside | 25 - 250 | $y = 4.52x10^{-5} – 0.06$ | 0.998 | 0.047 | 0.123 |
| 11 | Myricetin 3-*O*-glucoside | 25 - 250 | $y = 2.98x10^{-5} – 1.6$ | 0.989 | 0.027 | 0.081 |
